# Preparation and application of FNAOSiPPEA/Cu(II) as a novel magnetite almondshell based Lewis acid-Bronsted base nano-catalyst for the synthesis of pyrimidobenzothiazoles

**DOI:** 10.1186/s13065-022-00838-6

**Published:** 2022-06-11

**Authors:** Dina Mallah, Bi Bi Fatemeh Mirjalili

**Affiliations:** Department of Chemistry, College of Science, Yazd University, P.O. Box 89195-741, Yazd, Iran

**Keywords:** Nano-almondshell, Bifunctional nano-catalyst, Lewis acid/ Bronsted base, Organometallic catalyst, Pyrimidobenzothiazole

## Abstract

**Background:**

The magnetic nano-catalysts improve the contact between substrates and catalyst considerably and simple isolation of catalyst from reaction mixture. In this study, Fe_3_O_4_@nano-almondshell@OSi(CH_2_)_3_/2-(1-piperazinyl)ethylamine/Cu(II) abbreviated (FNAOSiPPEA/Cu(II)), was prepared, characterized and applied for the synthesis of 4*H*-pyrimido[2,1-*b*]benzothiazole.

**Results:**

FNAOSiPPEA/Cu(II) as a bio-based nano-catalyst was prepared from the complexation of copper on 2-(1-piperazinyl)ethylamine, which was immobilized on Fe_3_O_4_@nano-almondshell@OSi(CH_2_)_3_ section. This new heterogeneous bifunctional Lewis acid/Bronsted base catalyst (FNAOSiPPEA/Cu(II)) was characterized by various techniques such as FT-IR, FESEM, TGA, EDS-MAP, XRD, VSM, BET, TEM, and XPS. So, the catalytic performance of this recyclable nano-catalyst was determined to promote the synthesis of 4*H*-pyrimido[2,1-*b*]benzothiazole derivatives at 100 °C under solvent-free conditions.

**Conclusions:**

Magnetite nano-catalyst of (FNAOSiPPEA/Cu(II)) is easily separated by an external magnet and successfully reused up at least 3 times with a slight loss of yield of the desired product.

**Supplementary Information:**

The online version contains supplementary material available at 10.1186/s13065-022-00838-6.

## Introduction

4*H*-pyrimido[2,1-*b*]benzothiazoles [PBTs] are a class of fused-pyrimidine-containing heterocyclic skeletons that have a variety of medicinal applications, such as phosphodiesterase inhibition, anti-allergic, antitumor, anti-parkinsonism, antihypertensive, anti-inflammatory, anti-hypotensive, antimalarial, and antimicrobial [1–3]. Due to the desirable pharmacological importance and various application of these compounds, recently, many methods for synthesis of PBTs such as Fe_3_O_4_@nano-cell/Cu(II) [4], Nano-[Co-4CSP]Cl_2_ [5], Nano-Kaolin/Ti^4+^/Fe_3_O_4_ [6], iron fluoride [7], Fe_3_O_4_@NCs/Sb(V) [8], nano cellulose/BF_3_/Fe_3_O_4_ [9], Nano-Fe_3_O_4_@SiO_2_-TiCl_3_ [10], Nano-TiCl_2_/Cellulose [11], and Fe_3_O_4_@nano-cellulose/TiCl [12] have been reported. The development of new methods for the synthesis of these bioactive compounds is important.

Magnetite nanoparticles (MNPs) of Fe_3_O_4_ have much importance as recyclable magnetic catalysts in organic reactions [13–16]. The high polarity and surface charge of these MNPs cause them, usually condense to form large clusters, which this problem is usually solved relatively by coating on magnetic nanoparticles [17, 18]. In particular, metal complexes and stabilization of various metals in various oxidation states are valuable catalytic developments [19].

According to the world facing a pollution crisis in recent years, scientists and chemists are looking to discover materials that are not only environmentally friendly but also cost-effective and recyclable [20–22]. Recently, nanoscale catalysts have been widely utilized to accelerate the organic reaction in synthetic chemistry [23, 24] and display a higher catalytic performance than conventional heterogeneous catalysts [25, 26]. Especially, the benefits of catalysts become more pronounced when, firstly, they are based on a natural substance and, secondly, the recyclability problem is solved by attaching to a magnetic substrate. Due to the increasing public awareness of eco-friendly materials and natural resources, the use of renewable polymeric materials such as materials containing cellulose has attracted the attention of the scientific community [20]. Accordingly, almondshell can be used as one of the natural and available sources of cellulose for supporting volatile compounds. Nano-almondshell can be used as a shell for the protection of Fe_3_O_4_ nanoparticles.

**Fig. 1:**
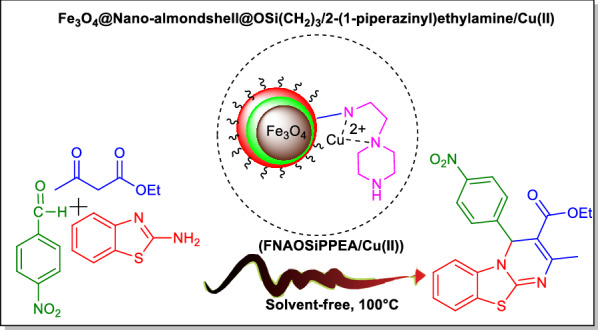
Application of (FNAOSiPPEA/Cu(II)) for the synthesis of PBT

2-(1-piperazinyl)ethylamine (2-PEA) that despite various biological activities can be used as a ligand for metal ions to form Lewis acid-Bronsted base catalysts [27]. In this research, a new magnetic double-layer bifunctional Lewis acid/Bronsted base catalyst named Fe_3_O_4_@Nano-almondshell@OSi(CH_2_)_3_/2-(1-piperazinyl)ethylamine/Cu(II) abbreviated (FNAOSiPPEA/Cu(II)) was prepared and characterized by FT-IR, FESEM, TGA, EDS-MAP, XRD, VSM, BET, TEM, and XPS. This novel catalyst was used for the synthesis of PBT derivatives (Fig. 1).

## Results and discussion

### Preparation of FNAOSiPPEA/Cu(II) nano-catalyst

**Fig. 2:**
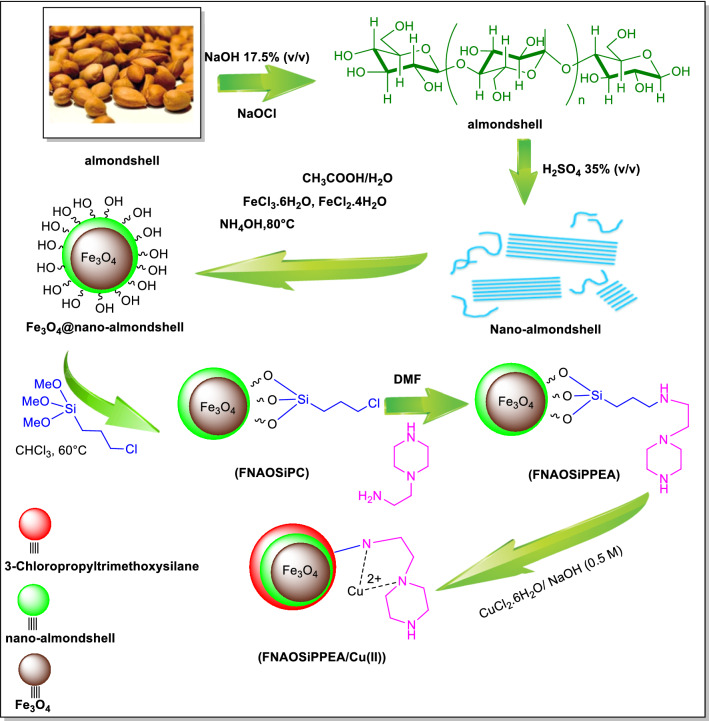
Preparation of FNAOSiPPEA/Cu(II)

A schematic representation of the sequence of events in the preparation of FNAOSiPPEA/Cu(II) is shown in Fig. 2. To the synthesis of the (FNAOSiPPEA/Cu(II)) with the Lewis acid/Bronsted base property, copper complexation on functional magnetite nano-almondshell. Initially, Fe_3_O_4_@nano-almondshell was prepared from nano-almondshell powder through previously reported methods for Fe_3_O_4_@nano-cellulose from cotton [12]. Then a mixture of Fe_3_O_4_@nano-almondshell and 3-(chloropropyl)-trimethoxysilane was refluxed for 4 h to prepare the Fe_3_O_4_@nano-almondshell@OSi(CH_2_)_3_Cl abbreviated (FNAOSiPC). Next, the dried FNAOSiPC reacted with 2-(1-piperazinyl)ethylamine in *N, N*-dimethylformamide under reflux conditions for 24 h to obtain Fe_3_O_4_@Nano-almondshell@OSi(CH_2_)_3_/2-(1-piperazinyl)ethylamine (FNAOSiPPEA). Finally, to immobilize Cu(II) and form (FNAOSiPPEA/Cu(II)), the CuCl_2_.2H_2_O solution was added to the FNAOSiPPEA at room temperature (Fig. 2). FT-IR, field emission scanning electron microscopy (FESEM), energy-dispersive X-ray spectroscopy (EDX), Thermogravimetric analysis (TGA), X-ray diffraction (XRD), Brunauer Emmett-Teller (BET) analysis, Transmission electron microscopy (TEM), and X-Ray Photoelectron Spectroscopy (XPS) analysis were used to identify the structure of the. FNAOSiPPEA/Cu(II) Additional file 1.

### FT-IR analysis of FNAOSiPPEA/Cu(II) nano-catalyst

**Fig. 3:**
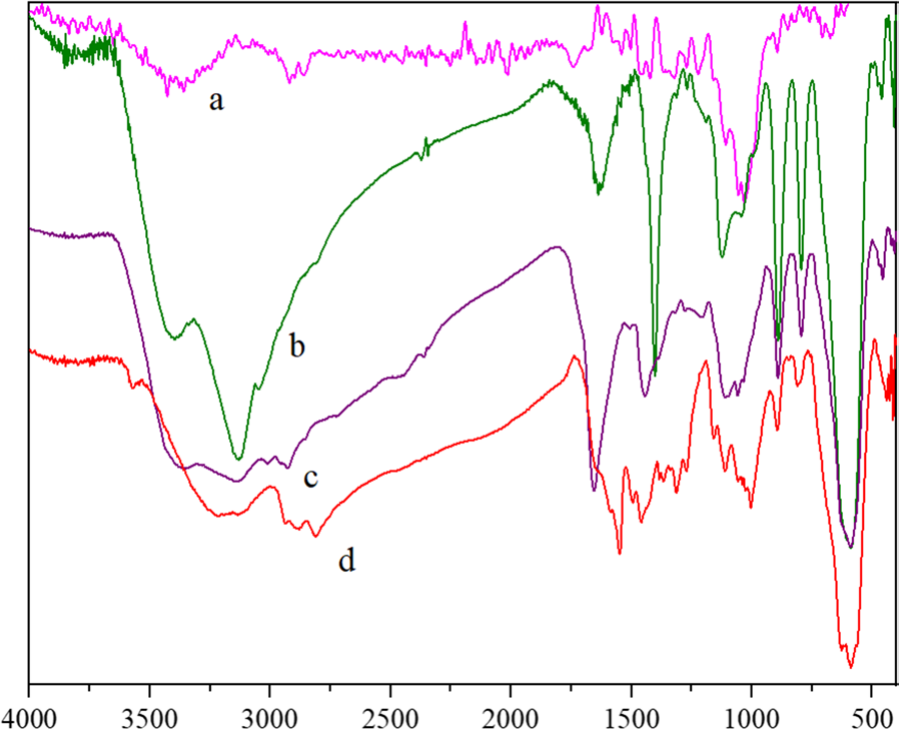
FT-IR spectra of (**a**) Nano-almondshell (**b**) Fe_3_O_4_@nano-almondshell (**c**) FNAOSiPPEA (**d**) FNAOSiPPEA/Cu(II)

The FT-IR spectra of almondshell, Fe_3_O_4_@nano-almondshell, FNAOSiPPEA, and FNAOSiPPEA/Cu(II) are shown in Fig. 3. Almondshell IR spectrum (Fig. 3a) shows the absorption peaks at 3428 cm^−1^, 2920 cm^−1^, and 1122 cm^−1^, which correspond to O–H, C-H, and C-O respectively. The absorption bands for Fe_3_O_4_@nano-almondshell (Fig. 3b) appeared at 3213 cm^−1^, 1122 cm^−1^, and 588 cm^−1^ for O-H, C-O, and Fe/O stretching vibrations. In (Fig. 3c), in addition to the absorption bands for almondshell and Fe_3_O_4_@nano-almondshell compounds, 1112 cm^−1^ and 1059 cm^−1^ are related to Si–O stretching vibrations, and indicate the presence of Si moiety in FNAOSiPPEA. The C-N stretching band in FNAOSiPPEA/Cu(II) (Fig. 3d) appears at 1432 cm^−1^, which is due to the complexation of Cu(II) in the catalyst.

### X-ray diffraction (XRD) analysis of FNAOSiPPEA/Cu(II) MNPs

**Fig. 4:**
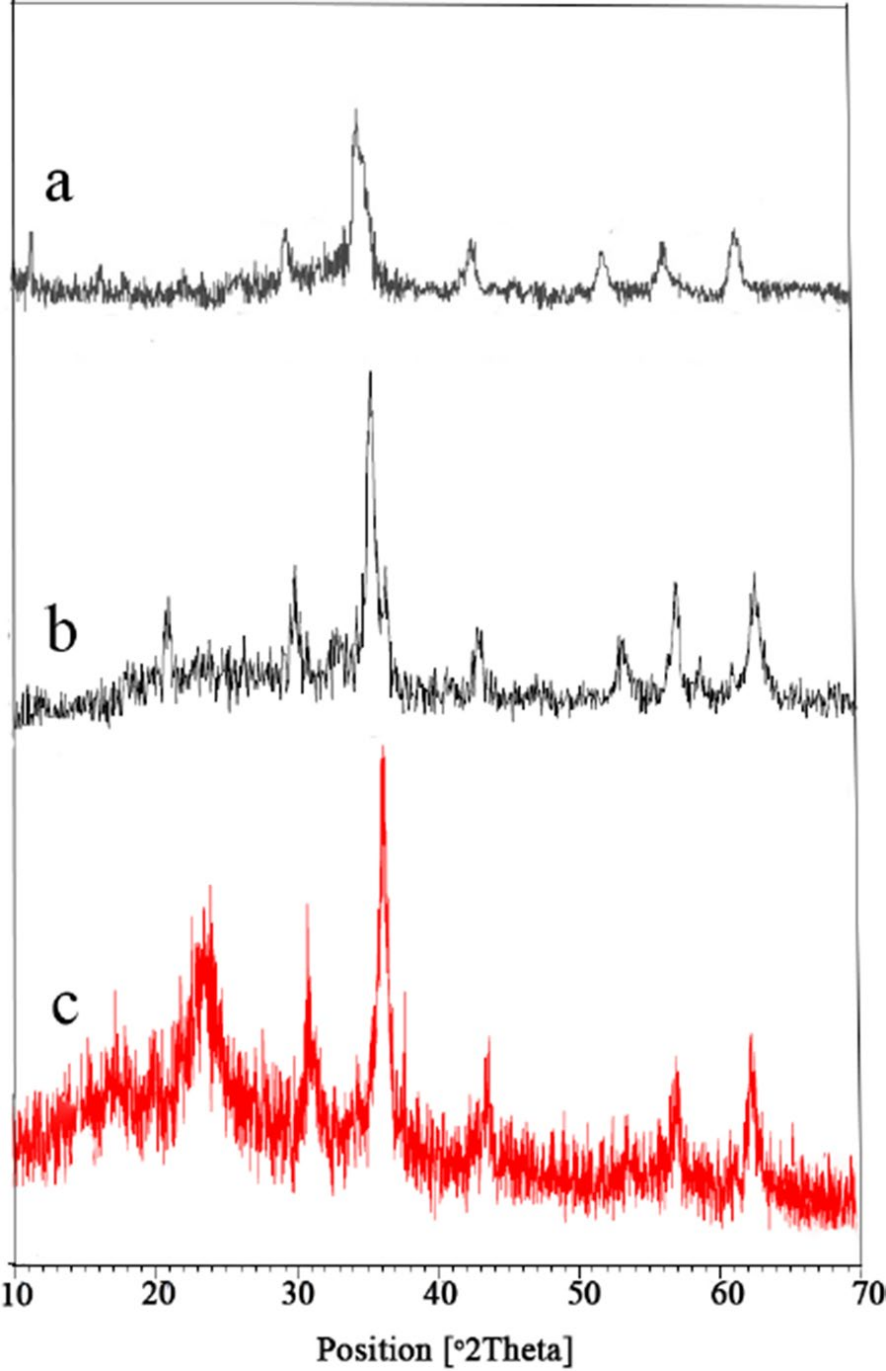
XRD diffraction patterns of (**a**) Fe_3_O_4_ (**b**) FNAOSiPPEA and (**c**) FNAOSiPPEA/Cu(II)

As shown in Fig. 4, the crystal structure of the initial Fe_3_O_4_ nanoparticles (Fig. 4a), and final FNAOSiPPEA/Cu(II) (Fig. 4c) was confirmed by XRD analysis. In the XRD pattern, several peaks appeared at 2*θ* = 31°, 35°, 43°, 54°, 57°, and 63° indicating that the original Fe_3_O_4_ crystalline structure was not destroyed. Besides, a peak at 2*θ* = 23° in FNAOSiPPEA and FNAOSiPPEA/Cu(II) has been appeared, which confirms the existence of almondshell in their structure and is formed around iron oxide NPs, as well as a broad peak at 2*θ* = 20–30° is related to amorphous silica (Fig. 4b, c). The weak peaks appear at 2*θ* = 38°, 43° reflection of the linked Cu (Fig. 4c).

### FESEM and TEM of FNAOSiPPEA/Cu(II)

**Fig. 5:**
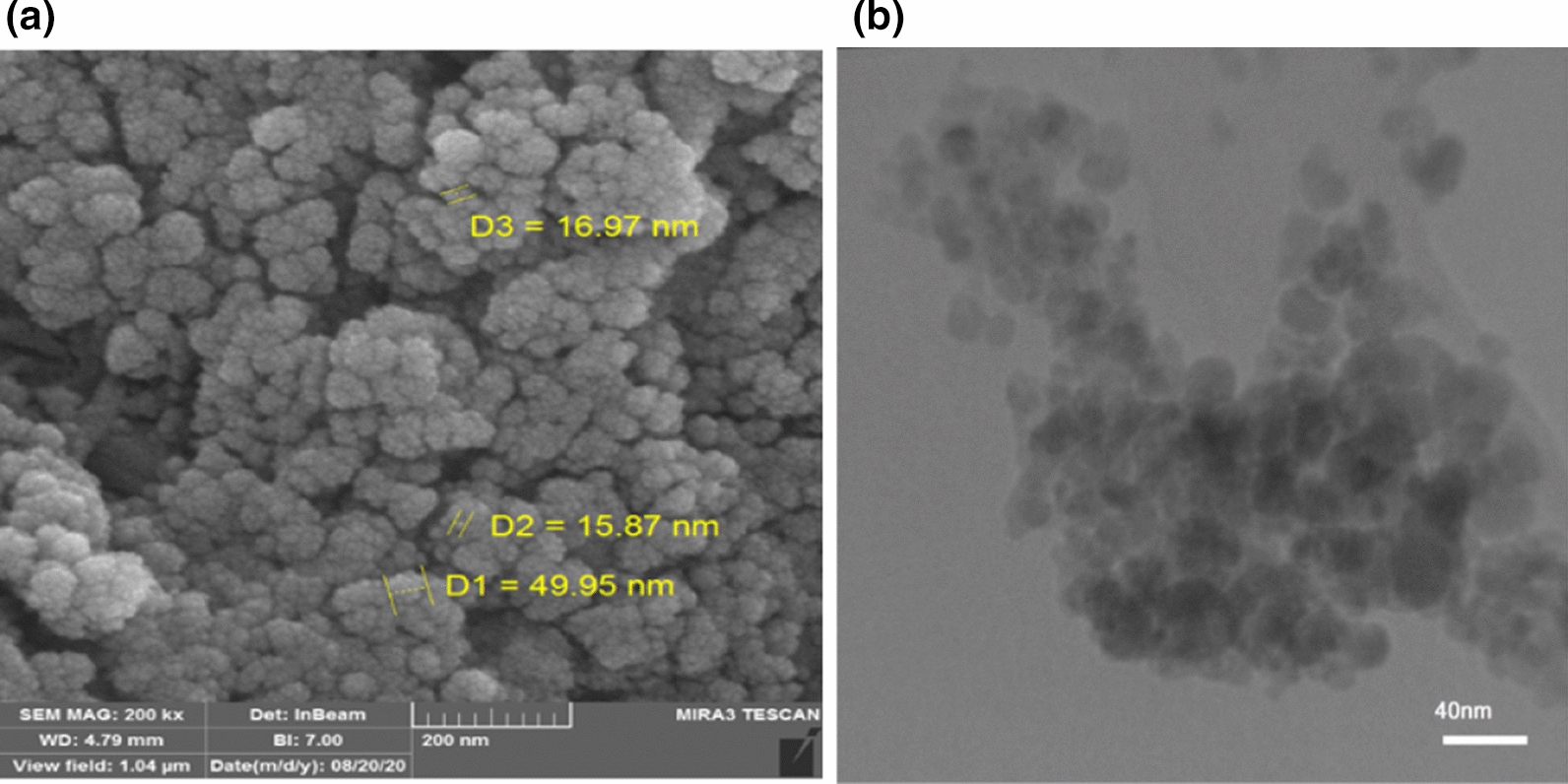
**a** FESEM image and **b** TEM of FNAOSiPPEA/Cu(II)

Figure 5a, Illustrates the particle size and surface morphology of the MNPs FNAOSiPPEA/Cu(II) using Field emission scanning electron microscopy (FESEM), the results show that the catalyst nanoparticles have a quasi-spherical shape, while the nanoparticles FNAOSiPPEA/Cu(II) still have nanoscale dimensions. Figure 5b, shows the Transmission electron microscopy **(**TEM) image of catalyst that approve the core–shell shape of it.

### Vibrating sample magnetization (VSM) of FNAOSiPPEA/Cu(II)

**Fig. 6:**
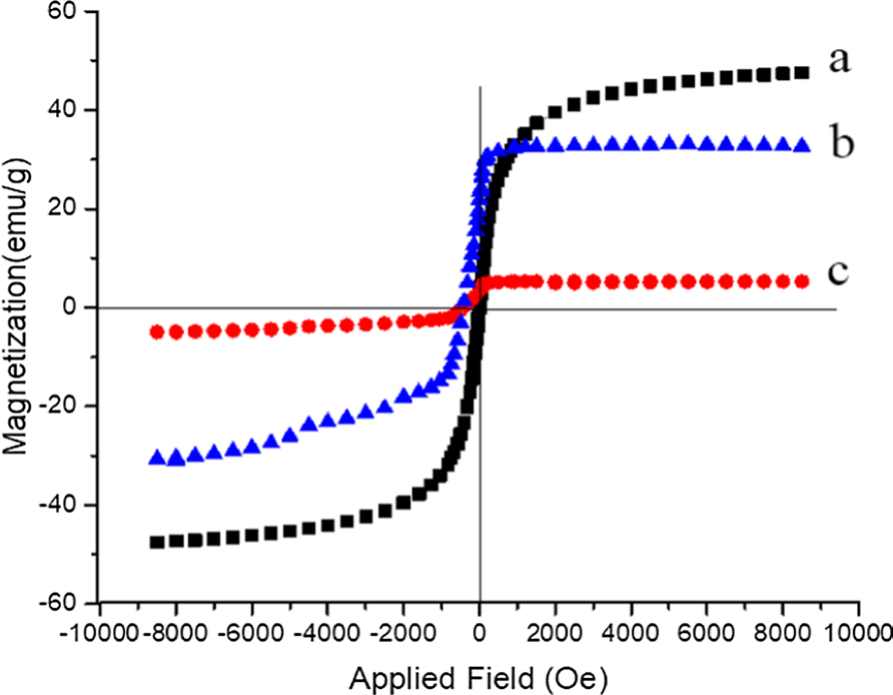
VSM analysis of (**a**) Fe_3_O_4_ NPs, **b** FNAOSiPPEA and **c** FNAOSiPPEA/Cu(II)

The magnetic properties of the FNAOSiPPEA/Cu(II) were evaluated by using the vibrating sample magnetometer (VSM) at room temperature. As Fig. 6 shows the zero coercivity and remanence of the hysteresis loops of these magnetic nanoparticles confirm the superparamagnetic property of them at room temperature. The value of magnetization of the samples varies from 47 to 33 emu g^−1^ after coating Fe_3_O_4_ with functional magnetite nano-almondshell and decreased to 5 emu g^−1^ after the complexation of Cu(II) on the surface of FNAOSiPPEA. The results show that the value of FNAOSiPPEA/Cu(II) saturation magnetization is less than nanoparticles Fe_3_O_4_, due to the formation of nano-almondshell and silica linker and the formation of Cu complex around the Fe_3_O_4_-core. Nevertheless, the magnetic nano-catalyst can still be easily separated from the reaction mixture by an external magnet.

### TGA analysis of FNAOSiPPEA/Cu(II) nano-catalyst

**Fig. 7:**
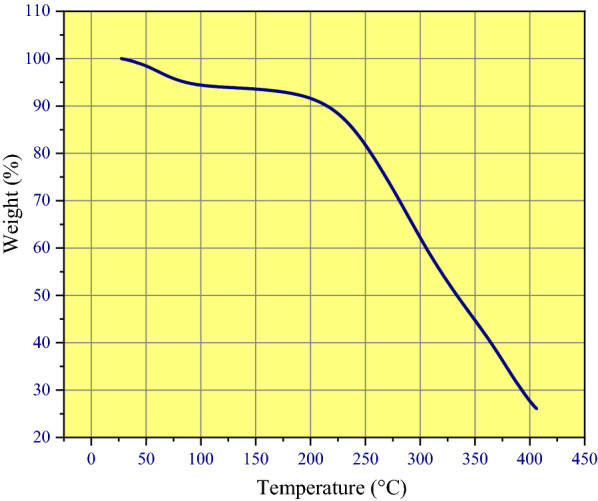
TGA diagram of FNAOSiPPEA/Cu(II)

The thermal stability of the FNAOSiPPEA/Cu(II), was determined by TGA in the temperature range of 50–400 ℃ (Fig. 7). The catalyst has almost two weight loss steps in the temperature range of 100–390 ℃. As can be seen, the TGA diagram shows the first weight loss (less than 100 ℃) can be related to the loss of surface adsorbed water and other solvents on the catalyst surface and the decomposition of its organic groups [28–30]. And the second in 180–370 ℃ is ascribed to the decomposition of almondshell. The exothermic process observed in the temperature range of 385–400 ℃, which leads to the third stage of weight loss, can be related to the Fe_3_O_4_ NPs decomposition [31]. The desired catalyst can be used in reactions around or below 200 ℃.

### EDS-map and energy dispersive X-ray (EDX) of FNAOSiPPEA/Cu(II)

**Fig. 8:**
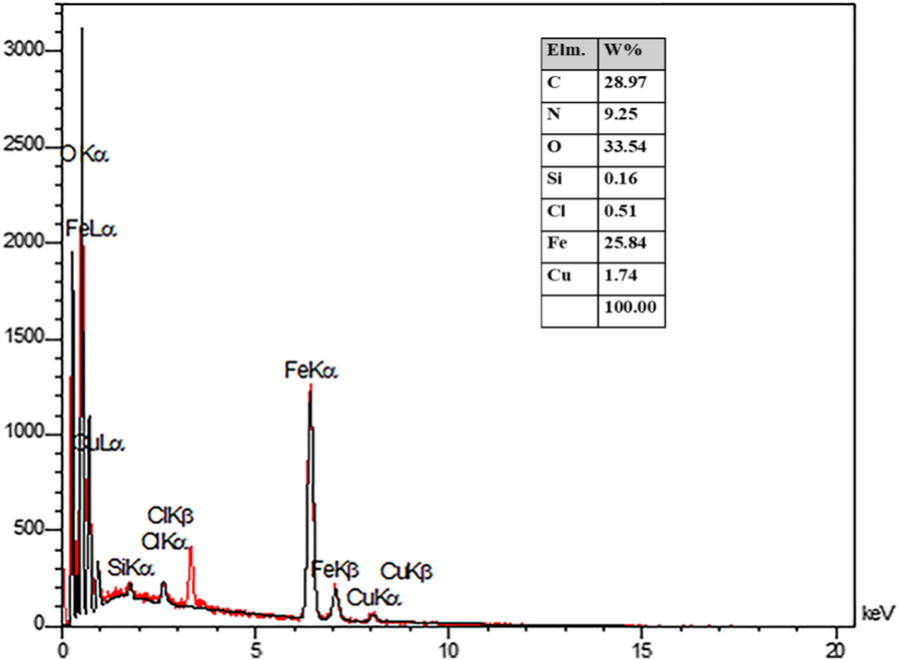
EDX analysis of FNAOSiPPEA/Cu(II)

**Fig. 9:**
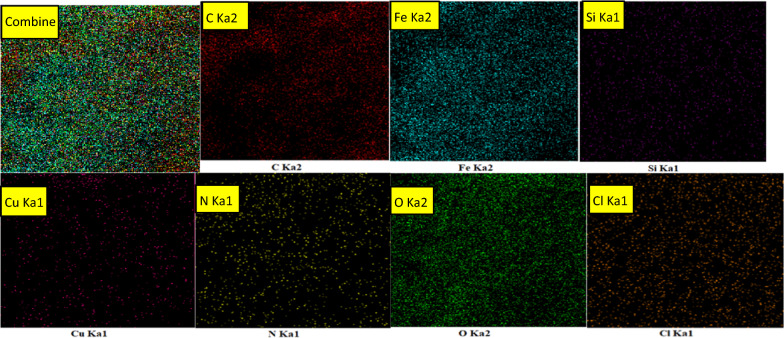
Element distribution maps showing the spatial distribution of each element

The composition elements of FNAOSiPPEA/Cu(II) were investigated by energy-dispersive X-ray spectroscopy (EDX). As Fig. 8 shows, the chemical properties of the prepared catalyst are composed of O 33.54%, C 28.97%, Fe 25.84%, N 9.25%, Cu 1.74%, Cl 0.51%, and Si 0.16% elements, which corresponds to the expected elements in the FNAOSiPPEA/Cu(II). The mapped SEM micrographs were obtained by using EDS-SEM analysis of the FNAOSiPPEA/Cu(II) to detect the composition of chemical elements on or in the surface catalyst (Fig. 9) The mapped SEM show, where the elements Fe (sky blue), O (green) and C (red) are well dispersed in the catalyst, while Si (purple), N (yellow) and Cl (orange) dominantly remain on the surface, and pink dots known as Cu is finely scattered on the surface of FNAOSiPPEA/Cu(II).

### Burunauer-emmett-taller (BET) analysis of FNAOSiPPEA/Cu(II) MNPs

**Fig. 10:**
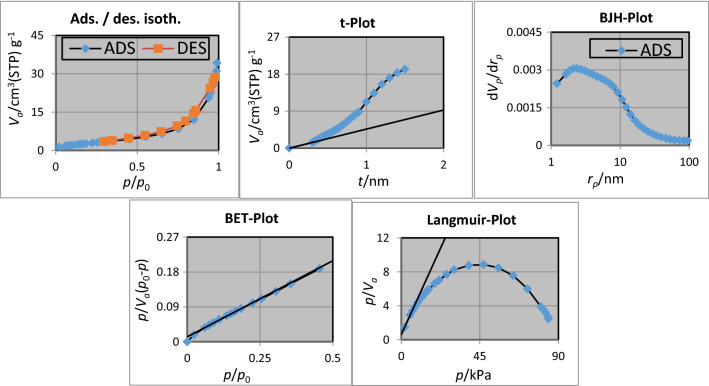
Diagrams calculated from the porosity measuring device

The nitrogen adsorption–desorption method is a valuable technique used to determine the specific surface area, volume, and distribution of pores. Nitrogen adsorption was measured on gas-depleted samples at 77 K. The adsorption–desorption isotherm (Fig. 10) is the type of II with H_3_ type hysteresis loop for FNAOSiPPEA/Cu(II), which is consistent with the IUPAC classification.

As can be shown in Table 1 the BJH (Barrett–Joyner–Halenda model) and pore diameters were 0.0541 cm^3^ g^−1^ and 19.501 nm respectively and the total pore volume is 0.0523 cm^3^ g^−1^, which in fact, modifying the surface of FNAOSiPPEA/Cu(II) reduces the pore space.

**Table 1:** Parameters obtained from porosity analysis

BET plot	
*V*_*m*_	2.4678[cm^3^(STP) g^−1^]
a_s,BET_	10.741[m^2^ g^−1^]
*C*	31.526
Total pore volume(*p*/*p*_0_ = 0.990)	0.052365[cm^3^ g^−1^]
Mean pore diameter	19.501[nm]
Langmuir plot	
*V*_*m*_	2.2129 [cm^3^(STP) g^−1^]
a_s,Lang_	9.6317 [m^2^ g^−1^]
B	0.7385
t plot	
Plot data	Adsorption branch
a_1_	7.1475 [m^2^ g^−1^]
V_1_	0 [cm^3^ g^−1^]
BJH plot	
Plot data	Adsorption branch
V_p_	0.054082 [cm^3^ g^−1^]
*r*_*p,peak*_(*Area*)	4.61[nm]
a_p_	15.62[m^2^ g^−1^]

### XPS analysis of FNAOSiPPEA/Cu(II) MNPs

**Fig. 11:**
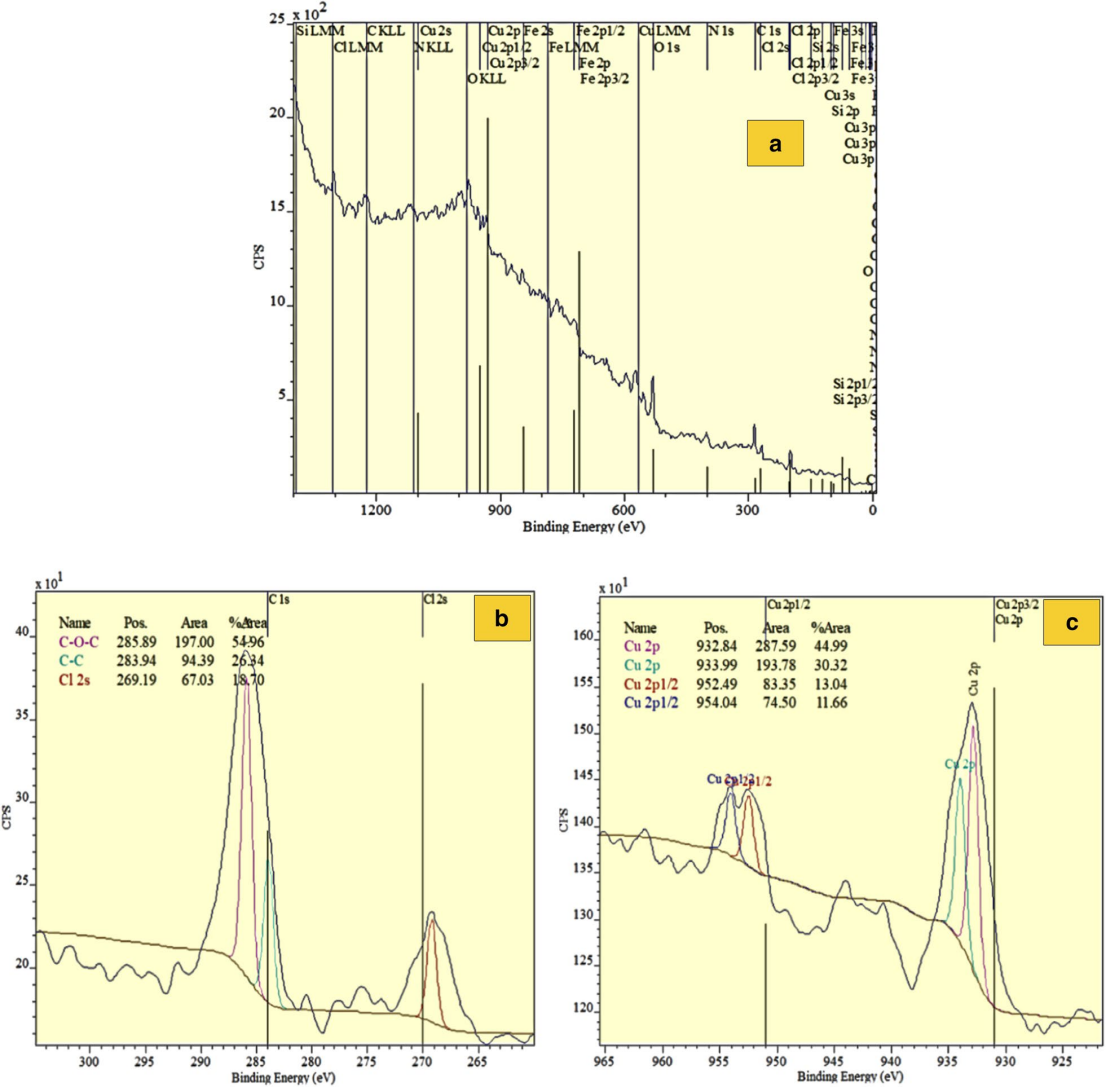

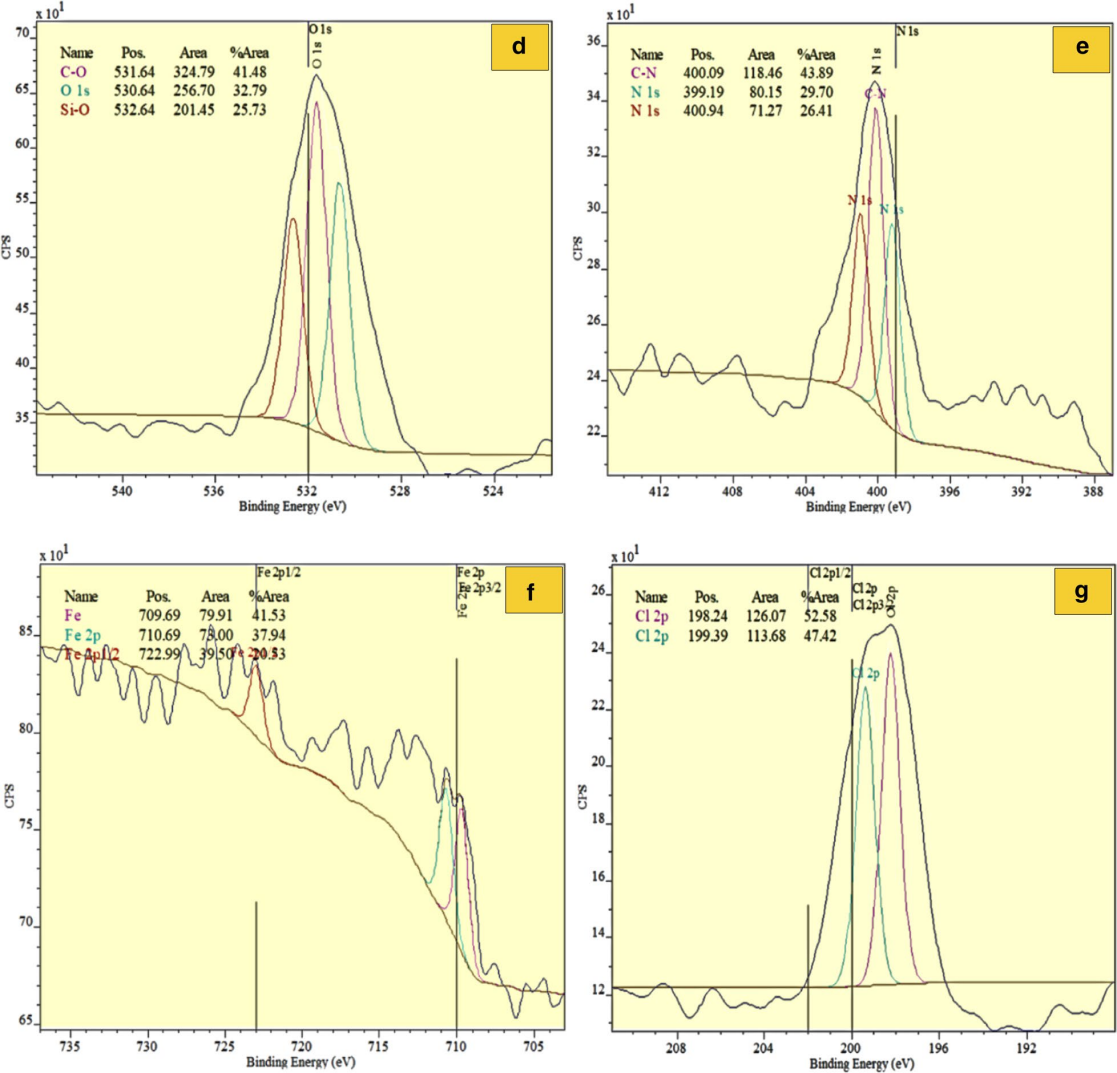
XPS spectrum of FNAOSiPPEA/Cu(II)

XPS analysis was used to investigate the coupling of Cu ions with FNAOSiPPEA (Fig. 11a–g). The C 1 s spectrum can be decomposed into two components around 283.94 eV and 285.94 eV (Fig. 11b). The first component with a binding energy of 283.94 eV is attributed to the carbon of the ring without oxygen (C–C) and the other component is attributed to the existing carbon of the C–O–C (285.89 eV). The peaks at around 932.84 eV and 933.99 eV in the Cu 2p spectrum correspond to the Cu(II) metal ion (Fig. 11c). This confirms peaks binding Cu(II) on the catalyst surface. The O 1 s spectrum was recorded for FNAOSiPPEA/Cu(II) (Fig. 11d). The peak at 531.64 eV was assigned to C-O oxygen, and the peak at 532.64 eV was assigned to Si–O. According to the N 1 s spectrum, the peak at 400.09 eV is attributed to C-N (Fig. 11e). The peaks appear at the binding energy of 709.69 eV and 198.24 eV are for Fe–O (Fig. 11f) and Cl 2p (Fig. 11g), respectively.

### Catalyst activity of FNAOSiPPEA/Cu(II)

**Fig. 12:**
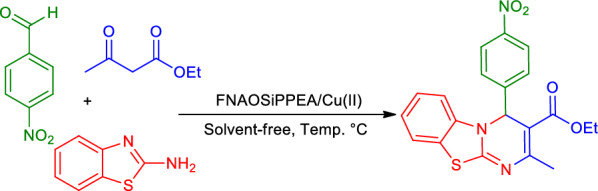
The model reaction for the synthesis of PBT under solvent-free conditions

Given the ability of nano-magnetic catalyst FNAOSiPPEA/Cu(II) as a recoverable, efficient, and Lewis acid/Bronsted base catalyst, we have decided to examine its catalytic performance for the synthesis of 4*H*-pyrimido[2,1-*b*]benzothiazole derivatives by three-component and one-pot reaction. Therefore, to optimize catalytic performance, we have studied the reaction of 4-nitrobenzaldehyde, 2-aminobenzothiazole, and ethyl acetoacetate (Fig. 12) in the molar ratio of 1:1:1 as a simple model reaction under different conditions. In order to, access optimal conditions, the amount of catalyst, initially, we have examined different amounts of catalyst in 25–100 ℃ under solvent-free conditions (Table 2).

**Table 2:** Optimization of amount catalyst and temperature for the synthesis of PBT under solvent-free conditions

Entry	FNAOSiPPEA/Cu(II) (g)	Temp. (℃)	Time (h)	Yield (%)^a^
1	–	25	7	30
2	–	100	3	32
3	0.03	80	5	47
4	0.03	100	3	56
5	0.04	25	6	32
6	0.04	50	4	40
7	0.04	80	4	61
8	0.04	90	2	83
9	0.04	100	1	97
10	0.04	110	2.5	90
11	0.05	80	3	48
12	0.05	100	4.5	57
13	0.07	100	6	33

Reaction conditions: 4-nitrobenzaldehyde (1 mmol), 2-aminobenzothiazole (1 mmol), and ethyl acetoacetate (1 mmol)

^a^Isolated Yield

**Fig. 13:**
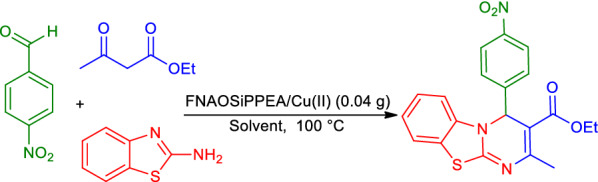
The model reaction for the synthesis of PBT using different solvents at 100 °C

The highest yield was obtained, when used 0.04 g of FNAOSiPPEA/Cu(II) at 100 ℃ and solvent-free conditions (Table 2, entry 9). We have also evaluated the effect of different temperatures on the model reaction, which increasing the reaction temperature, decreases reaction time at different values of the catalyst (Table 2, entry 9). Then, the model reaction was optimized in the presence of different solvents at 100 ℃ and 0.04 g of the FNAOSiPPEA/Cu(II) (Fig. 13, Table 3).

**Table 3:** Investigation of the effect of solvent on the synthesis of PBT at 100 ℃ in the presence of 0.04 g FNAOSiPPEA/Cu(II)

Entry	Solvent	Time (h)	Yield (%)^a^
1	–	1	97
2	EtOH	5	61
3	H_2_O	5	57
4	EtOH/H_2_O	5	48

Reaction conditions: 4-nitrobenzaldehyde (1 mmol), 2-aminobenzothiazole (1 mmol), and ethyl acetoacetate (1 mmol) under 100 ℃

^a^Isolated yield

As shown in Table 3, the reaction in solvent-free conditions results in high yields of the desired product.

**Fig. 14:**
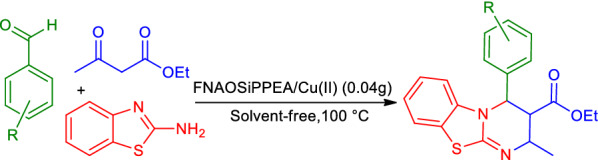
Synthesis of PBT under modified condition

Considering the achievement of optimal conditions for the model reaction in the solvent-free condition, to study the electronic and spatial nature of the substitute groups on the reactants, the reaction between 2-aminobenzothiazole, ethyl acetoacetate, and various aldehydes was investigated for the synthesis of PBT derivatives in the presence of 0.04 g FNAOSiPPEA/Cu(II) at 100 ℃ and solvent-free conditions (Fig. 14), which the results are summarized in Table 4.

**Table 4:** Synthesis of PBT derivatives in the presence of 0.04 g FNAOSiPPEA/Cu(II) at 100 ℃ and solvent-free conditions

Entry	R	Time (h)	Yield (%)^a^	m. p. (°C)	Refs.
1	H	1.15	85	177–179	[32]
2	4-NO_2_	1	97	171–173	[33]
3	4-Cl	1	95	86–88	[31]
4	4-Br	0.75	97	110–114	[31]
5	4-OH	1.30	84	210–212	[31]
6	2-Cl	2	90	122–123	[6]
7	2-OEt	2.5	89	171–173	[11]
8	2-NO_2_	1.5	79	122–124	[31]
9	3-NO_2_	1.20	95	222–223	[6]
10	3-OH	1.5	81	261–262	[6]
11	2,4-(Cl)_2_	1	87	133–134	[4]
12	2,4-(OMe)_2_	1.40	77	164–165	[12]
13	3,4-(OH)_2_	1.40	75	227–229	[6]

Reaction conditions: Aldehyde (1 mmol), 2-aminobenzothiazole (1 mmol), and ethyl acetoacetate (1 mmol), Solvent-free and 100 ℃

^a^Isolated yield

Investigation of the results in Table 4 shows that the reaction rate and yield in the synthesis of PBTs for aromatic aldehydes containing electron-withdrawing group are higher.

### Comparative of catalytic activity of FNAOSiPPEA/Cu(II) nano-catalyst

To show the efficiency and ability of this catalyst and other catalysts used in the synthesis of PBT through the three-component reaction model, 4-nitrobenzaldehyde, 2-aminobenzothiazole, and ethyl acetoacetate was compared and the results are tabulated in Table 5. The results clearly show that the present catalyst is more efficient both in terms of reaction time and efficiency. Besides, in the present method are used the least amount of catalyst and solvent-free conditions.

**Table 5:** Comparison of FNAOSiPPEA/Cu(II) with other catalysts for the synthesis of PBT

Entry	Amount of Catalyst (g)	Solvent	Temp. (℃)	Time (h)	Yield (%)	Refs,
1	Trypsin (0.03 g)	Ethylene glycol	60	48	94	[34]
2	Chitosan/HOAc (0.08 g)	H_2_O	60	1.40	72	[35]
3	SMI-SO_3_H (0.08 g)	Solvent-free	100	3	80	[36]
4	Nano-cellulose/BF_3_/Fe_3_O_4_ (0.06 g)	Solvent-free	100	1	80	[9]
5	Tetrabutylammonium hydrogen sulfate (10 mol %)	Ethylene glycol	120	2	83	[37]
6	FNAOSiPPEA/Cu(II) (0.04 g)	Solvent-free	100	1	97	This work

### Recyclability of FNAOSiPPEA/Cu(II) MNPs

**Fig. 15:**
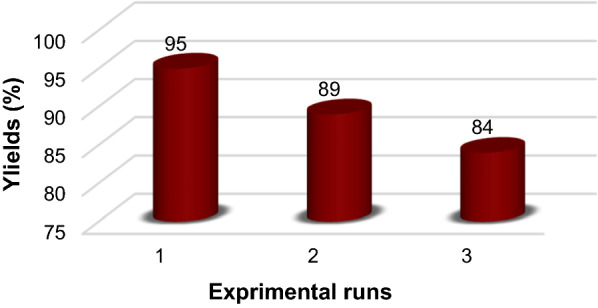
Reusability of FNAOSiPPEA/Cu(II) as a catalyst for the synthesis of PBT

To check the reusability of the catalyst, after completion of the model reaction under optimized conditions, ethanol was added to the reaction mixture and the catalyst was separated by an external magnet. The separated catalyst was washed with ethanol and dried at 60 ℃. The results show, that the recycled catalyst can be reused up to at least 3 times with a slight loss of catalytic activity under optimal conditions (Fig. 15).

### Proposed mechanism for the synthesis of 4*H*-pyrimido[2,1-*b*]benzothiazole derivatives

**Fig. 16:**
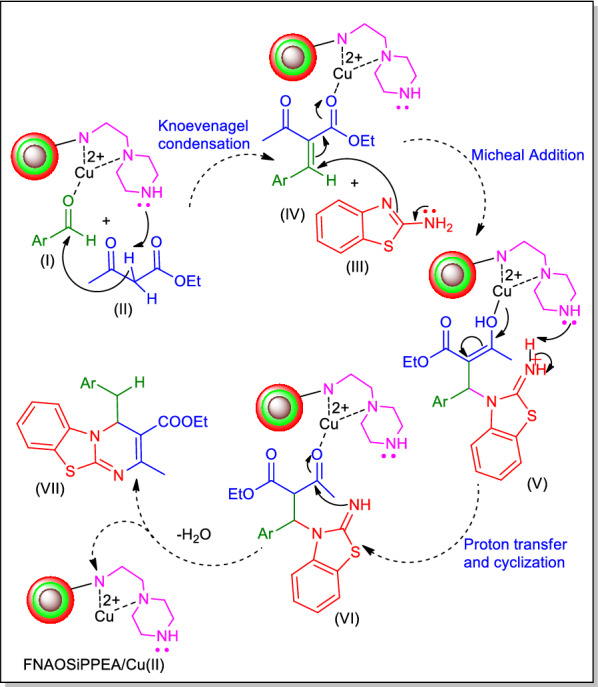
The possible mechanism for the synthesis of 4*H*-pyrimido[2,1-*b*]benzothiazole

The possible mechanism for the synthesis of 4*H*-pyrimido[2,1-*b*]benzothiazole derivatives is described in Fig. 16. The surface of the catalyst most likely consists of hydroxyl groups and the Cu complex acts as Lewis acid. According to the mechanism, first, the carbonyl group of aldehyde (I) is activated by the Cu complex immobilized on functional Fe_3_O_4_ NPs, then the alkene (IV) is formed via the Knoevenagel condensation from the reaction of the aldehyde (I) with *β*-ketoester (II). In the next step, the alkene (IV) with 2-aminobenzothiazole (III) forms (V) via the Michael addition, which produces the target product (VI) after proton transfer and intramolecular cyclization.

## Conclusion

In summary, we have investigated that the immobilization of copper (Cu(II)) on Fe_3_O_4_@almondshell@Si(CH_2_)_3_/2-(1-piperazine)ethylamine produces a bifunctional Lewis acid/ Bronsted base, recyclable, environmentally friendly, and bio-based catalyst. This catalyst is suitable for the synthesis of 4*H*-pyrimido[2,1-*b*]benzothiazole derivatives under 100 ℃ and solvent-free conditions. Easy workup, short reaction time, and excellent yield of products are some advantages of the present protocol.

## Experimental

### Materials and methods

Chemicals were purchased from Merck, Fluka, and Aldrich Chemical Companies. ^1^H NMR and ^13^C NMR spectra were recorded at 400 and 100 MHz, respectively. Fourier transform infrared (FT-IR) measurements (in KBr pellets or ATR) were recorded on a Brucker spectrometer. Melting points were determined on a Büchi B-540 apparatus. The X-ray diffraction (XRD) pattern was obtained by a Philips XpertMPD diffractometer equipped with a Cu Kα anode (*k* = 1.54 Å) in the 2*θ* range from 10 to 80˚. Field Emission Scanning Electron Microscopy (FESEM) was obtained on a Mira 3-XMU. VSM measurements were performed by using a vibrating sample magnetometer (Meghnatis Daghigh Kavir Co. Kashan Kavir, Iran). Energy-dispersive X-ray spectroscopy (EDS) of nano-catalyst was measured by an EDS instrument and Phenom pro X. The EDX-MAP micrographs were obtained on MIRA II detector SAMX (France). Thermal gravimetric analysis (TGA) was conducted using the “STA 504” instrument. BELSORP MINI II nitrogen adsorption apparatus (japan) for recording Brunauer–Emmett–Teller (BET) of nano-catalyst at 77 K [4, 12]. X-Ray Photoelectron Spectroscopy (XPS) analysis was done with BESTEC (EA 10). Transmission electron microscopy (TEM) was obtained using a Philips CM120 with a LaB6 cathode and accelerating voltage of 120 kV.

### Preparation of nano-almondshell

To prepare the nano-almondshell, the almondshell was heated in boiling water for 30 min, dried, and powdered. The next was treated with a 17.5 w/v NaOH solution at 90 ℃ for 24 h under reflux conditions. Subsequently, the almondshell was filtered and washed with distilled water until the alkali was eliminated. Then, bleached with 100 mL of 1:1 aqueous dilution of 3.5% w/v sodium hypochlorite (NaOCl) at 80 ℃ for 3 h under reflux conditions. The resulting almondshell particles were hydrolyzed partially using 35% sulfuric acid (H_2_SO_4_) aqueous solution with an almondshell-to-acid weight ratio of 1 to 10 at 45 ℃. After 3 h, the obtained suspension was diluted with water five-fold to stop the hydrolysis reaction. The suspension was centrifuged at 4000 rpm to separate the nano-almondshell from the acid solution (yield 60%).

### Preparation of Fe_3_O_4_@nano-almondshell

In a 250 mL flask, 3 g of nano-almondshell and 100 mL acetic acid (CH_3_COOH) of 0.05 M were added. Then FeCl_3_.6H_2_O (3.51 g, 13 mmol) and FeCl_2_.4H_2_O (1.29 g, 6.5 mmol) were added and stirred for 6 h at 80 °C. After that, 8 mL of NH_4_OH (25%), was added drop wise and stirred for 45 min. Then, the precipitated brown products were isolated from the solution by an external magnet, washed 3 times with distilled water, and dried in an oven at 80 ℃ for 4 h. The weight of the Fe_3_O_4_@nano-almondshell obtained is 4.141 g.

### Synthesis of FNAOSiPC

In a 100 mL flask, 1 g of dried Fe_3_O_4_@nano-almondshell was dispersed in the mixture of 10 mL of chloroform, and 3.4 mL 3-chloropropyltrimethoxysilane was added dropwise. The mixture was sonicated at 25 °C for 20 min and then, the mixture was carried out under reflux conditions for 4 h. The result was decanted using an external magnet and washed three times with chloroform.

### Synthesis of FNAOSiPPEA

The FNAOSiPC (0.5 g) was dispersed in ethanol by ultrasonic for 20 min at room temperature and then dried. After that, 0.5 g of dried FNAOSiPC and 2-(1-piperazine-yl) ethylamine (0.129 mL, 1 mmol) was heated in 10 mL N*, N*-dimethylformamide (DMF) under reflux condition for 24 h at 80 °C. The resulting precipitates were cooled and washed with dichloromethane (CH_2_Cl_2_) and then dried.

### Preparation of FNAOSiPPEA/Cu(II)

In a round bottom flask containing 50 mL of 0.5 M NaOH, functionalized-FNAOSiPC (0.5 g) was added with stirring. Subsequently, 75 mL of CuCl_2_ aqueous solution, 0.04 M, was added and stirred at room temperature. After 6 h, the magnetic FNAOSiPPEA/Cu(II) was separated from the mixture by a magnet. The catalyst was washed with ethanol and water and dried in an oven at 80 ℃.

### General procedure for the synthesis of 4*H*-pyrimido[2,1*-b*]benzothiazole derivatives

A mixture of 2-aminobenzothiazole (1 mmol), aldehyde (1 mmol), ethyl acetoacetate (1 mmol), and FNAOSiPPEA/Cu(II) (0.04 g) was stirred at 100 ℃ under solvent-free condition. After completion of the reaction (monitored by TLC, n-hexane: EtOAc [7:3]), the mixture was dissolved in hot ethanol (5 mL) and the catalyst was separated by an external magnet. Then, by adding drop-wise water (1 mL) to the reaction mixture, the precipitates of the product appeared as a pure solid in high yield.

## Supplementary Information

**Additional file 1.** Spectroscopic data for the synthesized 4H-Pyrimido[2,1-b]benzothiazole derivatives.

## Data Availability

All data generated or analyzed during this study are included in this published article.

## Abbreviations

PBT: 4*H*-Pyrimido[2,1-*b*]benzothiazole
MNPs: Magnetite nanoparticles
2-PEA: 2-(1-Piperazinyl)ethylamine
NPs: Nanoparticles
NaOH: Sodium hydroxide
NaOCl: Sodium hypochlorite
H_2_SO_4_: Sulfuric acid
CH_3_COOH: Acetic acid
NH_4_OH: Ammonium hydroxide
CH_3_Cl: Chloroform
DMF: *N*, *N*-Dimethylformamide
CH_2_Cl_2_: Dichloromethane
EtOAc: Ethyl acetate
EtOH: Ethanol
FESEM: Field emission scanning electron microscopy
EDX: Energy-dispersive X-ray spectroscopy
TGA: Thermogravimetric analysis
XRD: X-ray diffraction
BET: Brunauer Emmett-Teller
