# Supplementary material for: Preparation and application of FNAOSiPPEA/Cu(II) as a novel magnetite almondshell based Lewis acid-Bronsted base nano-catalyst for the synthesis of pyrimidobenzothiazoles
Source: BMC Chem. 2022 Jun 11;16(1):45. doi: 10.1186/s13065-022-00838-6 (PMC9188727; doi:10.1186/s13065-022-00838-6)
Supplement: Supplementary file 1 — Additional file 1. Spectroscopic data for the synthesized 4H-Pyrimido[2,1-b]benzothiazole derivatives. [file 13065_2022_838_MOESM1_ESM.pdf]

Preparation and application of FNAOSiPPEA/Cu(II) as a novel magnetite almondshell based Lewis acid-Bronsted base nano-catalyst for the synthesis of pyrimidobenzothiazoles

Dina Mallah and Bi Bi Fatemeh Mirjalili

Department of Chemistry, College of Science, Yazd University, Yazd, P.O.Box 89195-741, I.R.Iran, E-mail:fmirjalili@yazd.ac.ir, Telephone: +983531232672, Fax: +98 3538210644

Ethyl-2-methyl-4-(phenyl)-4*H*-pyrimido[2,1-*b*][1,3]benzothiazole-3-carboxylate

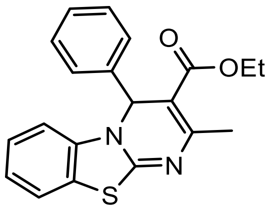

Pale yellow solid. ^{1}H NMR (DMSO-d_6, 400 MHz): δ 7.73 (d, *J*=7.7 Hz, 1H), 7.41 (t, *J*=7 Hz, 2H), 7.27-7.34 (m, 4H), 7.16-7.24 (m, 2H), 6.46 (s, 1H), 4.02-4.08 (m, 2H), 2.33 (s, 3H), 1.17 (t, *J* = 7 Hz, 3H). IR (KBr): 2972, 1669, 1592, 1460, 1241, 748 cm^{-1}. mp: 177-179 $^{\circ}$C.

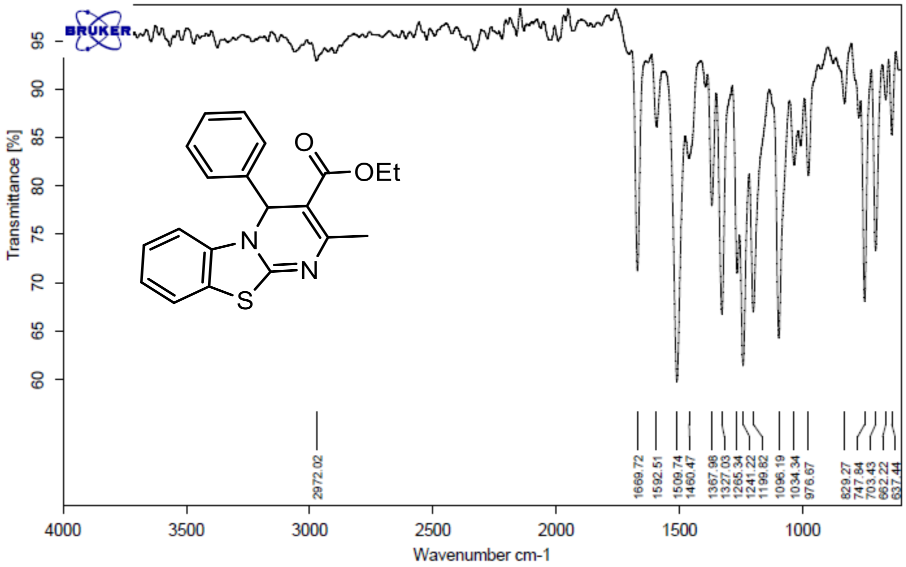

S1: The FT-IR spectrum of Ethyl-2-methyl-4-(phenyl)-4*H*-pyrimido[2,1-*b*][1,3]benzothiazole-3-carboxylate

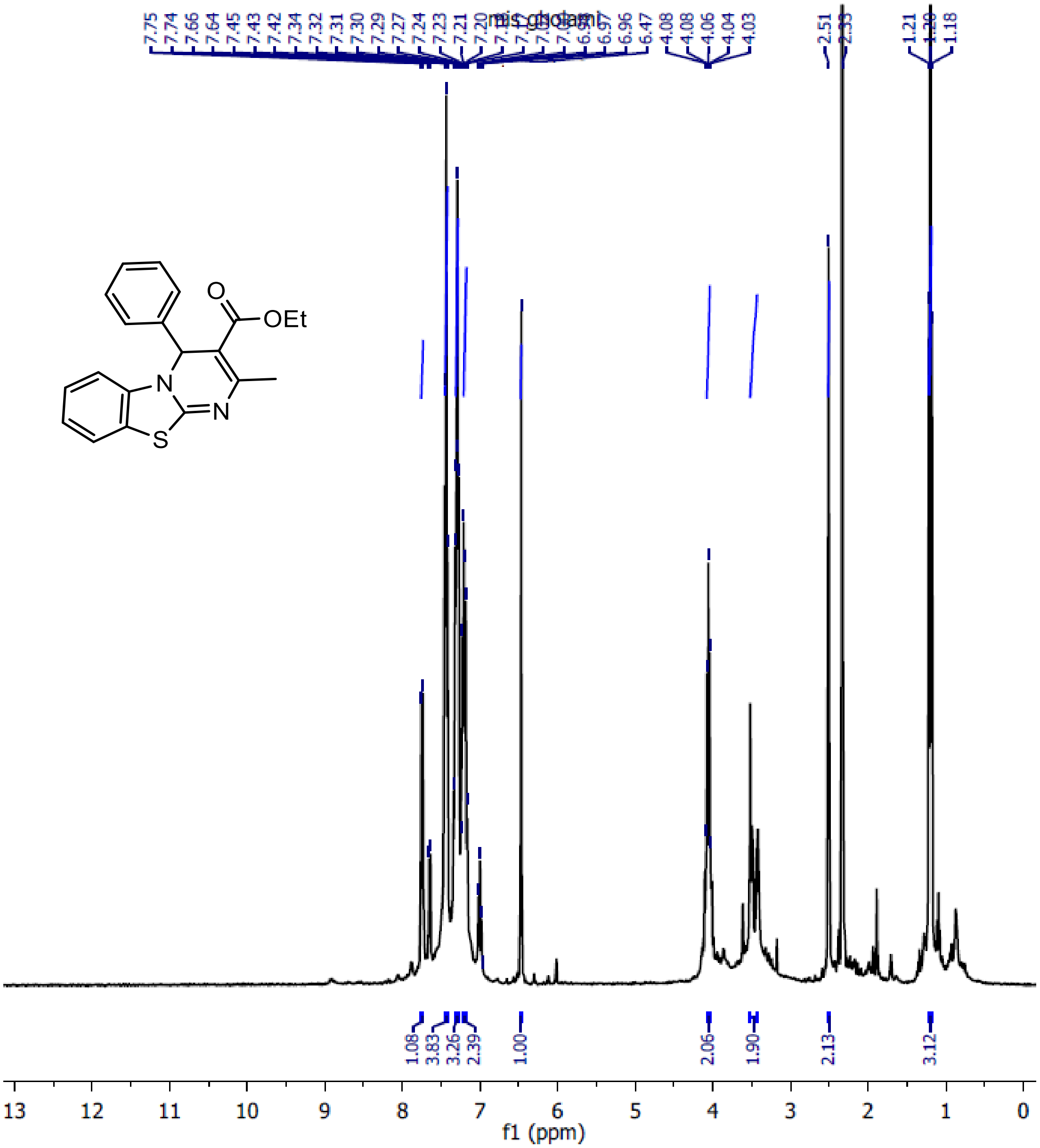

S2. The ^{1}H NMR (400MHz) spectrum of Ethyl-2-methyl-4-(phenyl)-4*H*-pyrimido[2,1-*b*][1,3]benzothiazole-3-carboxylate

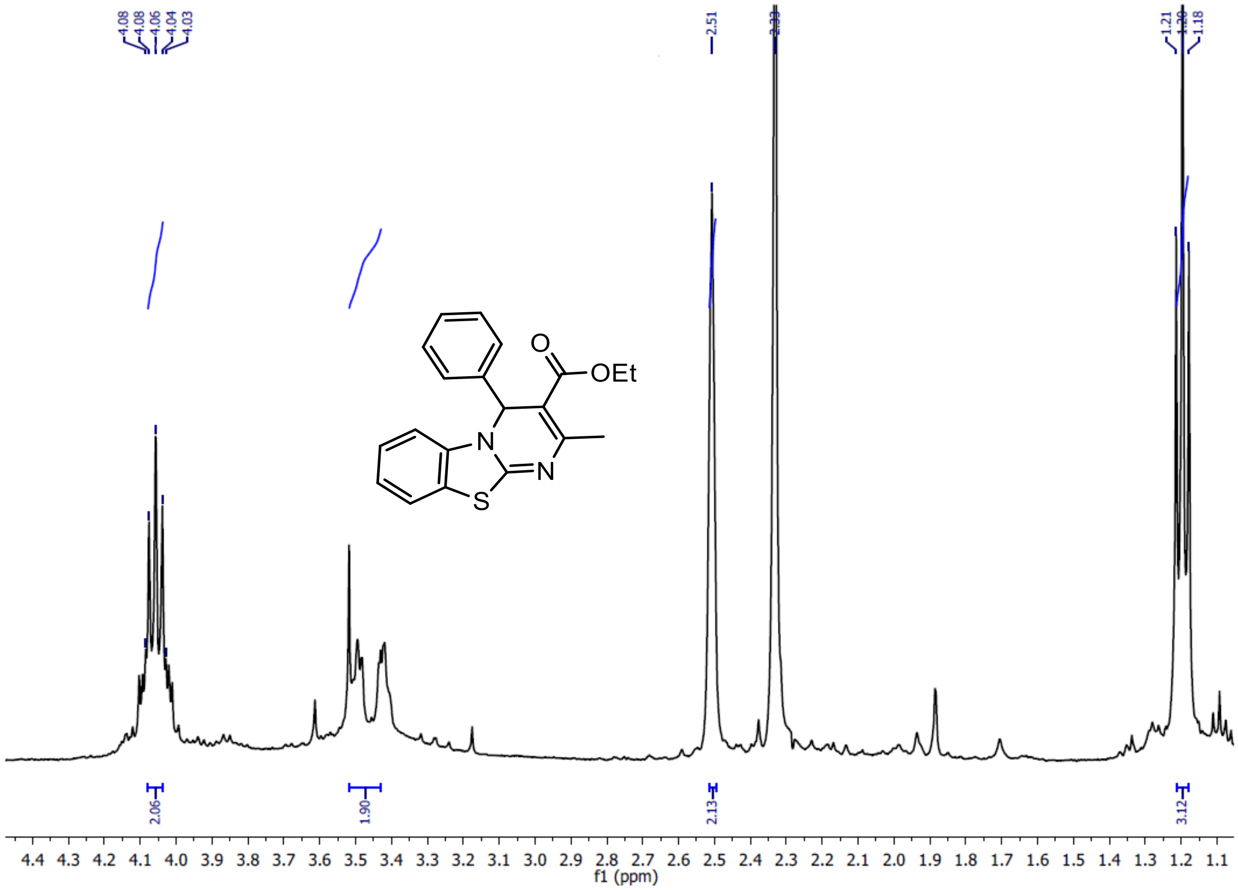

S3. The ^{1}H NMR (400MHz) spectrum of Ethyl-2-methyl-4-(phenyl)-4*H*-pyrimido[2,1-*b*][1,3]benzothiazole-3-carboxylate

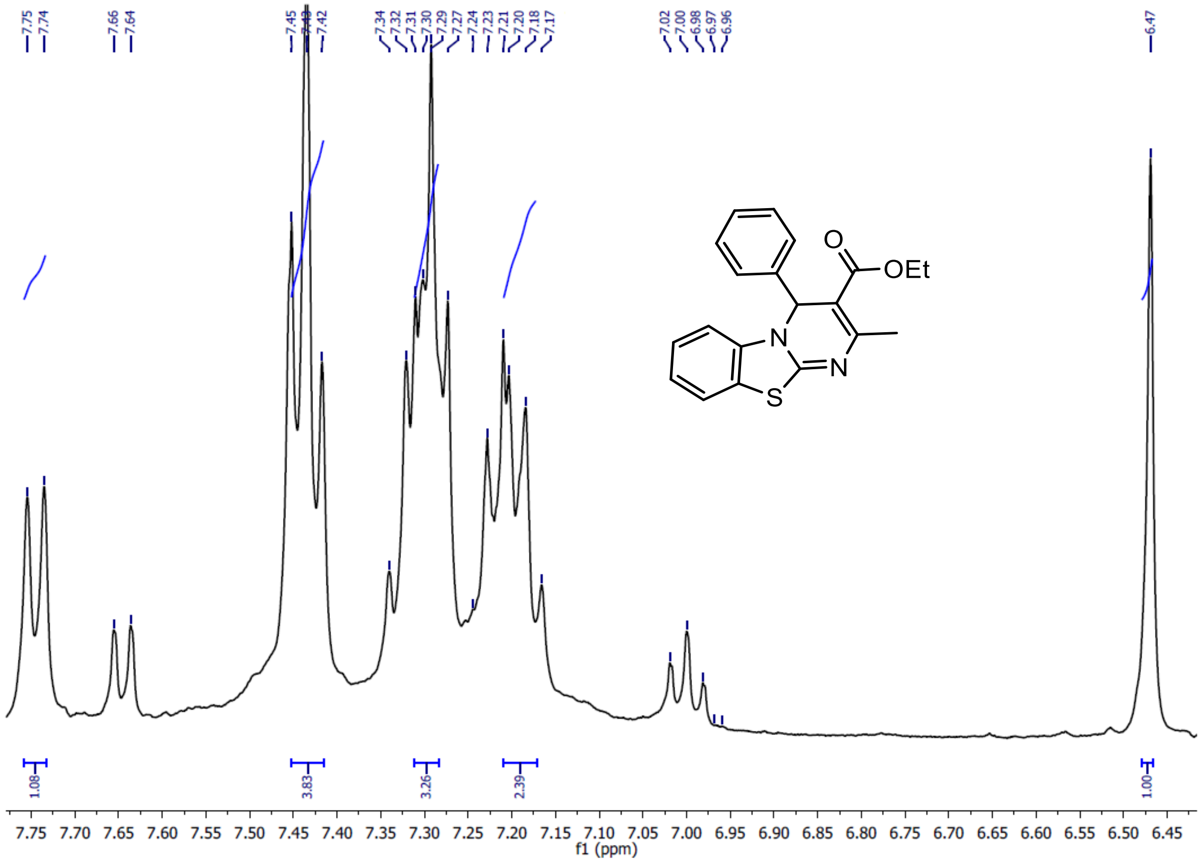

S4. The ^{1}H NMR (400MHz) spectrum of Ethyl-2-methyl-4-(phenyl)-4*H*-pyrimido[2,1-*b*][1,3]benzothiazole-3-carboxylate

Ethyl-2-methyl-4-(4-nitrophenyl)-4*H*-pyrimido[2,1-*b*][1,3]benzothiazole-3-carboxylate

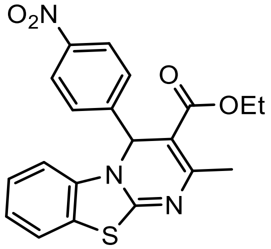

Yellow solid. ^{1}H NMR (DMSO-d$_6$, 400 MHz): δ 8.17 (br. s, 2H), 7.75 (br. s, 3H), 7.47 (br. s, 1H), 7.31 (br. s, 1H), 7.21 (br. s, 1H), 6.66 (s, 1H), 4.06 (br. s, 2H), 2.37 (s, 3H), 1.21 (br. s, 3H). IR (KBr): 2979, 1690, 1580, 1491, 1344, 1275, 1241, 1199, 746 cm^{-1}. mp: 171-173 $^{\circ}$C.

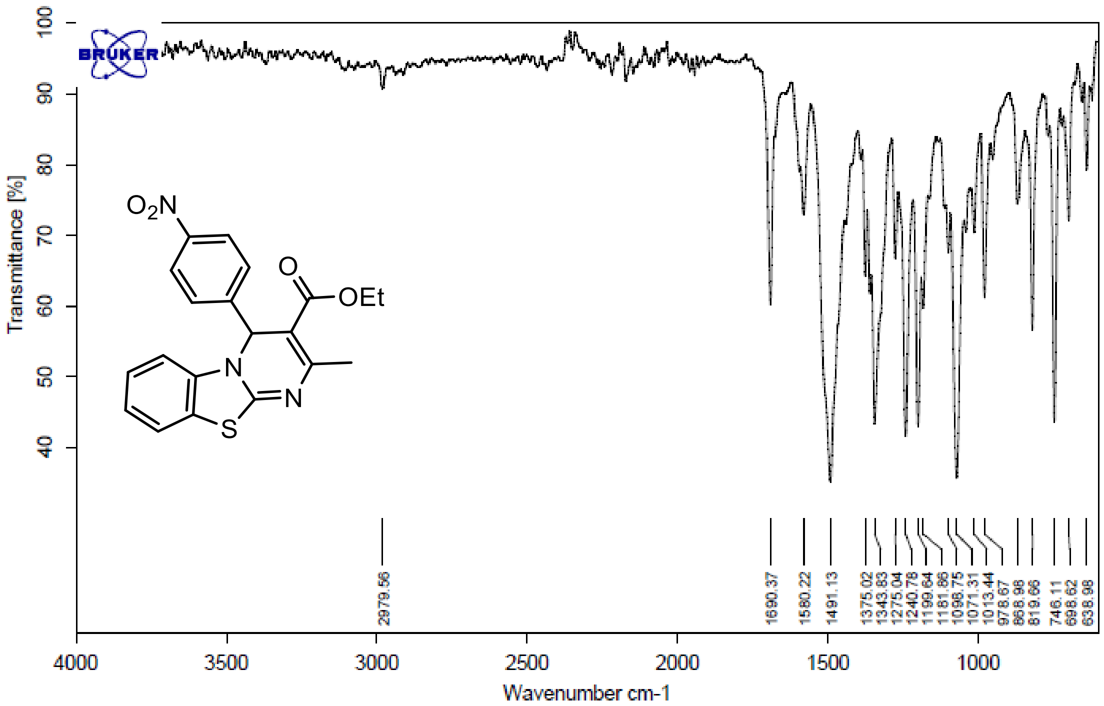

S5. The FT-IR spectrum of Ethyl-2-methyl-4-(4-nitrophenyl)-4*H*-pyrimido[2,1-*b*][1,3]benzothiazole-3-carboxylate

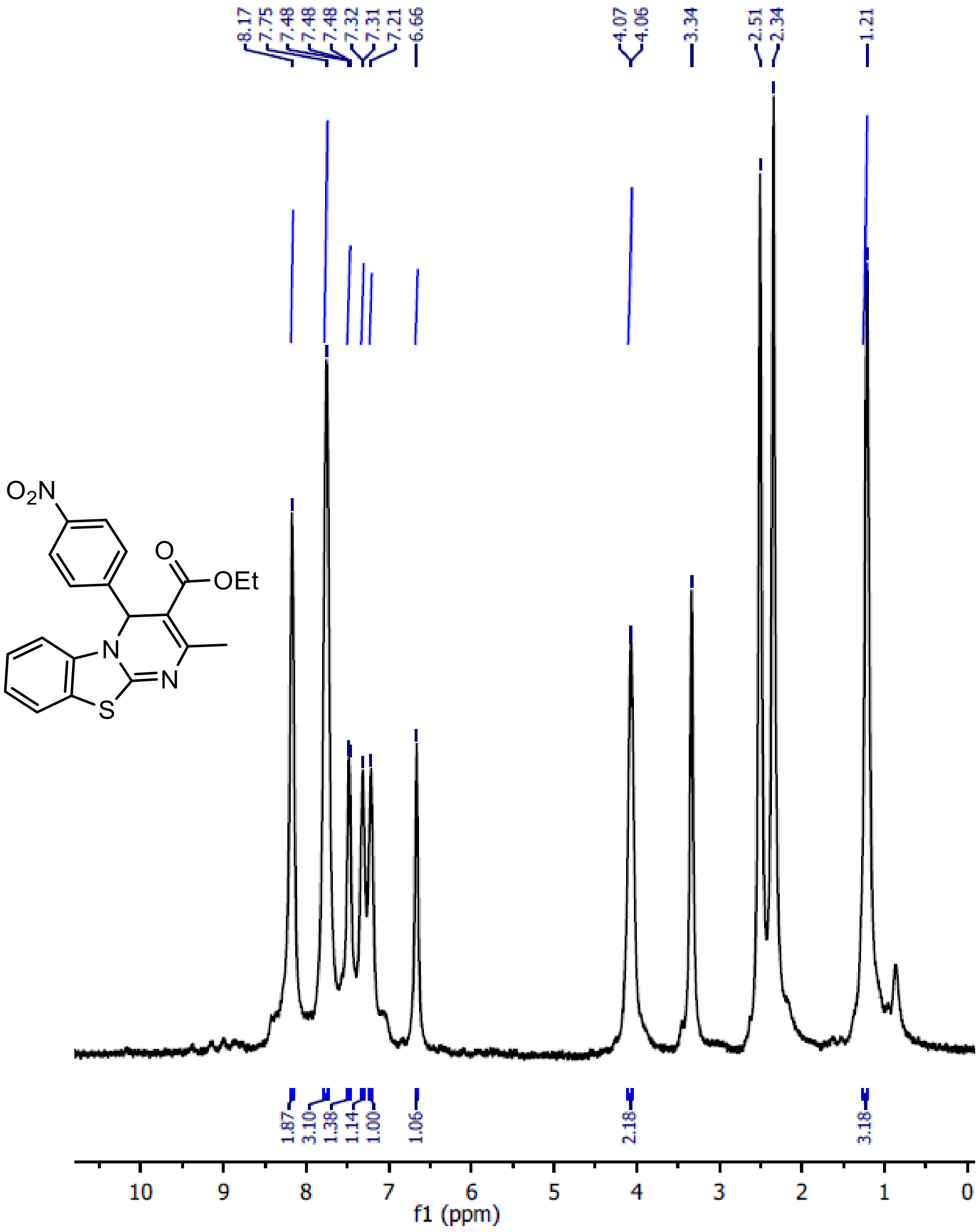

S6. The ^{1}H NMR (400MHz) spectrum of Ethyl-2-methyl-4-(4-nitrophenyl)-4*H*-pyrimido[2,1-*b*][1,3]benzothiazole-3-carboxylate

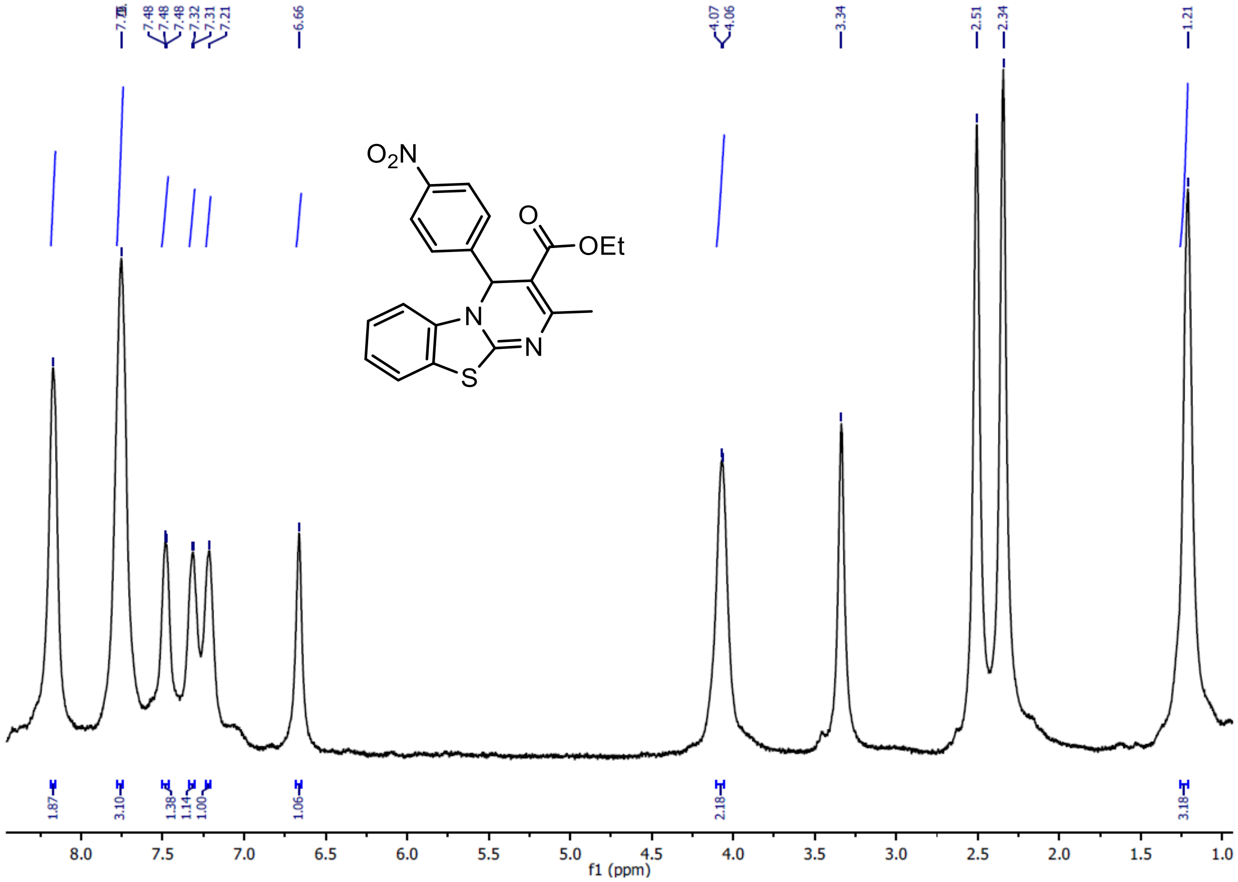

S7. The ^{1}H NMR (400MHz) spectrum of Ethyl-2-methyl-4-(4-nitrophenyl)-4*H*-pyrimido[2,1-*b*][1,3]benzothiazole-3-carboxylate

Ethyl-2-methyl-4-(4-chlorophenyl)-4*H*-pyrimido[2,1-*b*][1,3]benzothiazole-3-carboxylate

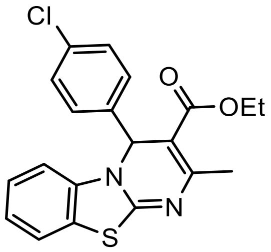

Yellow solid. ^{1}H NMR (DMSO-d$_6$, 400 MHz): δ 7.75 (br. s, 1H), 7.46-7.50 (m, 2H), 7.32-7.37 (m, 4H), 7.20 (br. s, 1H), 6.49 (s, 1H), 4.04-4.06 (t, J = 4.5 Hz, 2H), 2.5 (s, 3H), 1.10-1.30 (s, 3H). IR (KBr): 2978, 1687, 1579, 1488, 1239, 1200, 1075, 831, 743 cm^{-1}. mp: 86-88 °C.

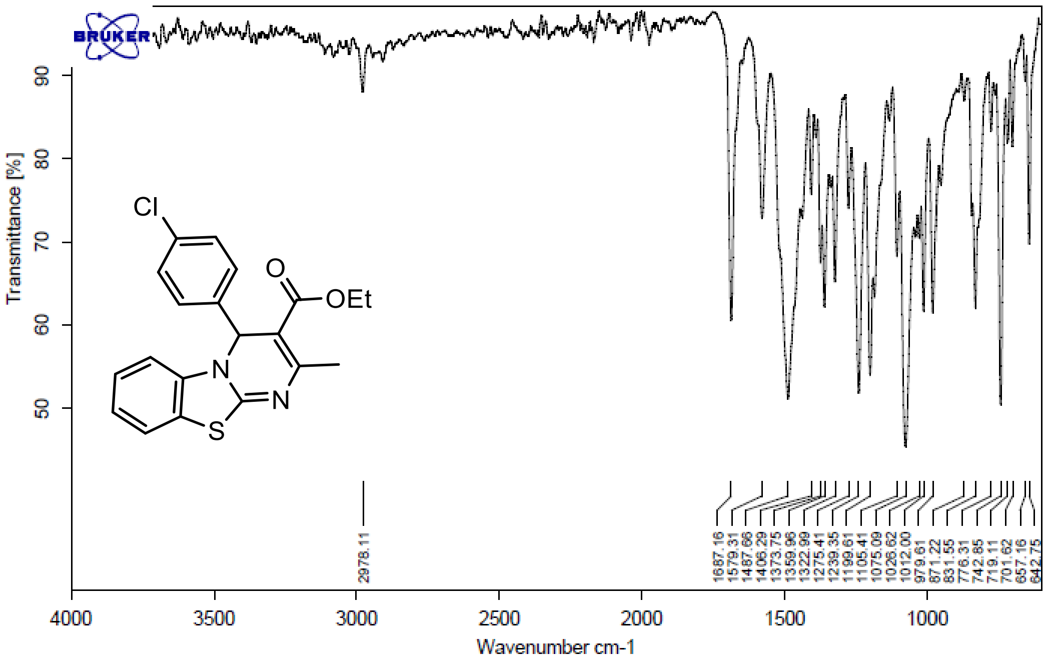

S8. The FT-IR spectrum of product of Ethyl-2-methyl-4-(4-chlorophenyl)-4*H*-pyrimido[2,1-*b*][1,3]benzothiazole-3-carboxylate

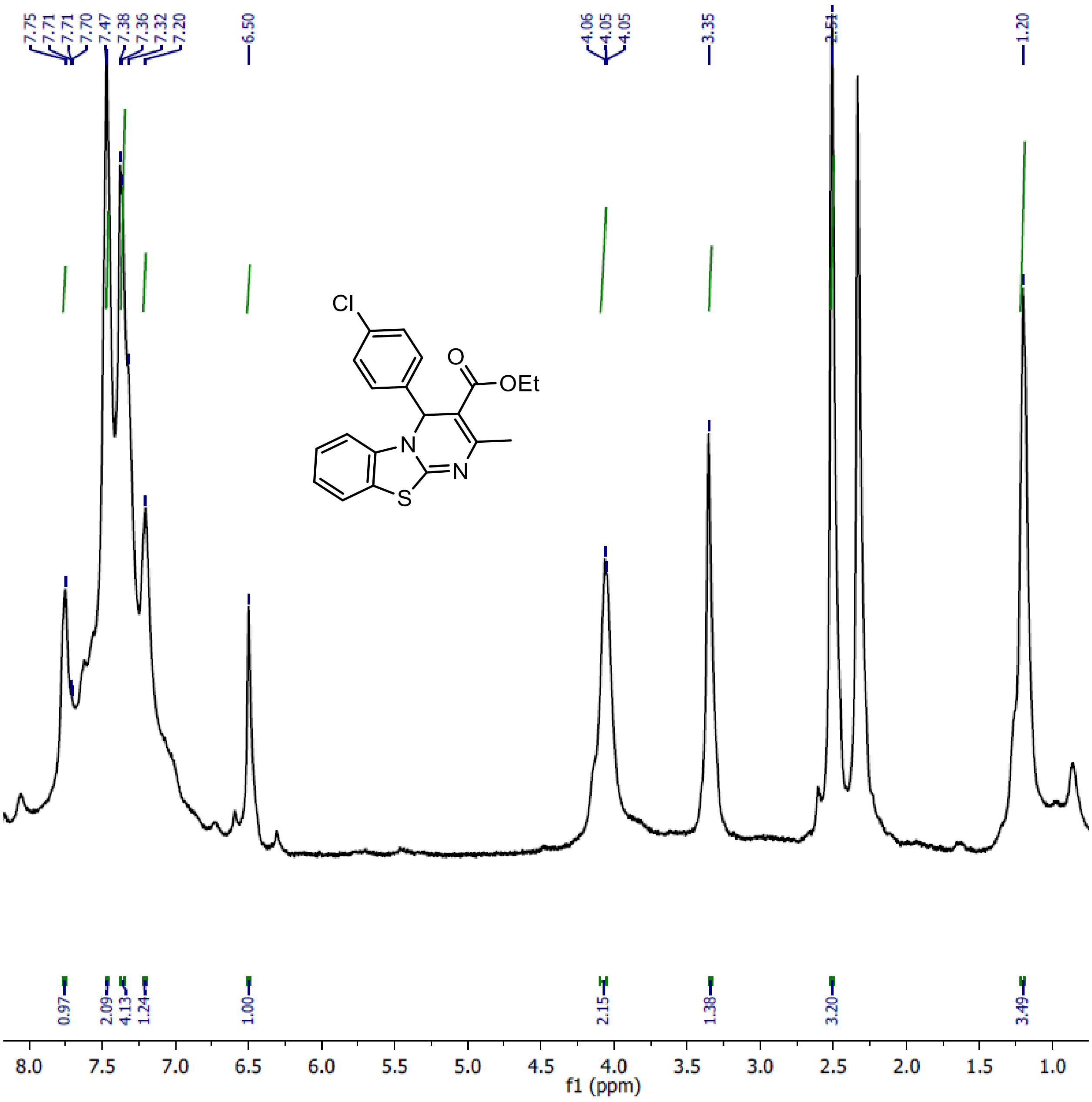

S9. The ^{1}H NMR (400MHz) spectrum of Ethyl-2-methyl-4-(4-chlorophenyl)-4*H*-pyrimido[2,1-*b*][1,3]benzothiazole-3-carboxylate

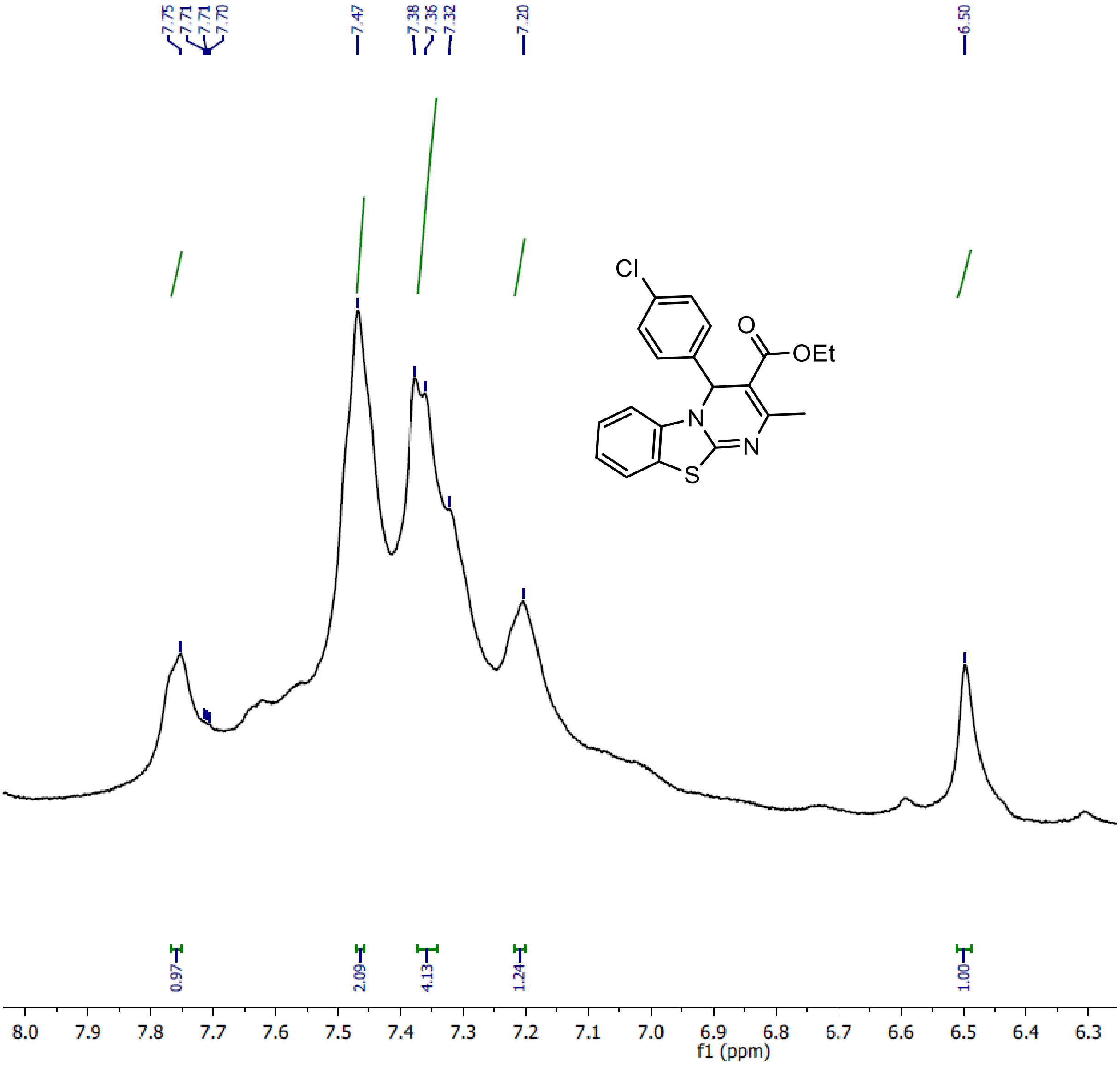

S10. The ^{1}H NMR (400MHz) spectrum of Ethyl-2-methyl-4-(4-chlorophenyl)-4*H*-pyrimido[2,1-*b*][1,3]benzothiazole-3-carboxylate

Ethyl-2-methyl-4-(4-bromo phenyl)-4*H*-pyrimido[2,1-*b*][1,3]benzothiazole-3-carboxylate

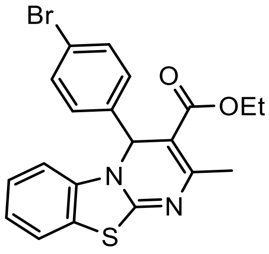

Orange solid. ^{1}H NMR (DMSO-d$_6$, 400 MHz): δ 7.75 (d, *J*=6.2 Hz, 1H), 7.40-7.51 (m, 5H), 7.21 (t, *J*= 6 Hz 1H), 6.52 (s, 1H), 4.04-4.08 (t, *J*=7 Hz, 2H), 2.50 (s, 3H), 1.10 (t, *J*= 6.8 Hz, 3H). IR (KBr): 2978, 1695, 1669, 1584, 1486, 1361, 1326, 1269, 1237, 1200, 1069, 1009, 832, 741 cm^{-1}. mp: 110-114 °C.

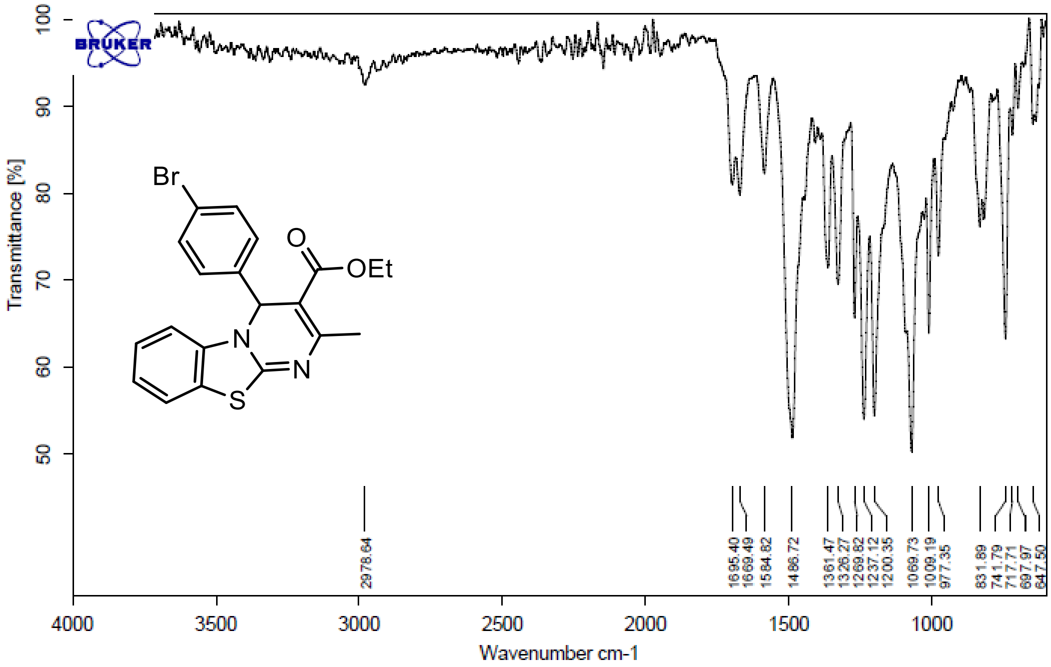

S11. The FT-IR spectrum of Ethyl-2-methyl-4-(4-bromo phenyl)-4*H*-pyrimido[2,1-*b*][1,3]benzothiazole-3-carboxylate

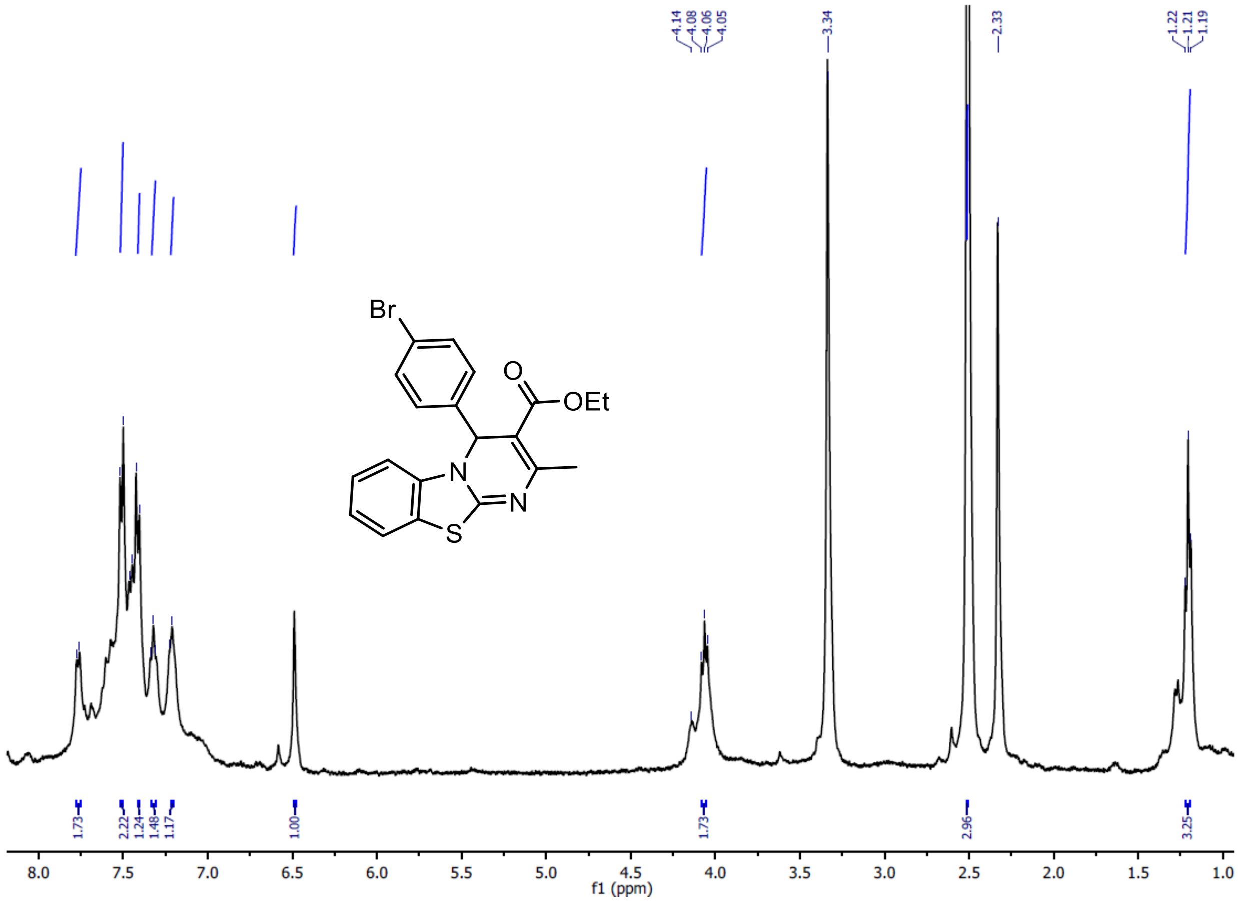

S12. The ^{1}H NMR (400MHz) spectrum of Ethyl-2-methyl-4-(4-bromo phenyl)-4*H*-pyrimido[2,1-*b*][1,3]benzothiazole-3-carboxylate

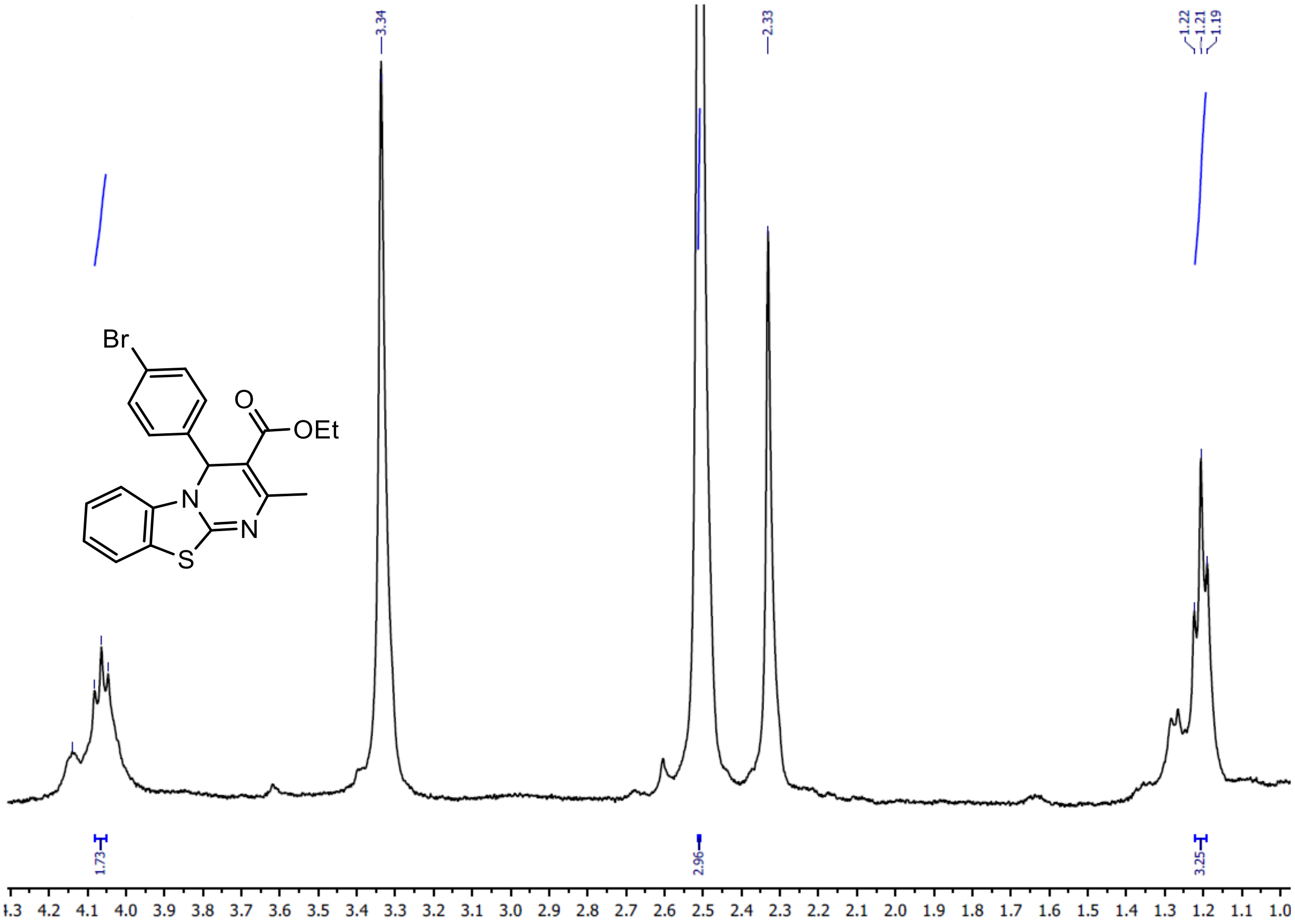

S13. The ^{1}H NMR (400MHz) spectrum of Ethyl-2-methyl-4-(4-bromo phenyl)-4*H*-pyrimido[2,1-*b*][1,3]benzothiazole-3-carboxylate

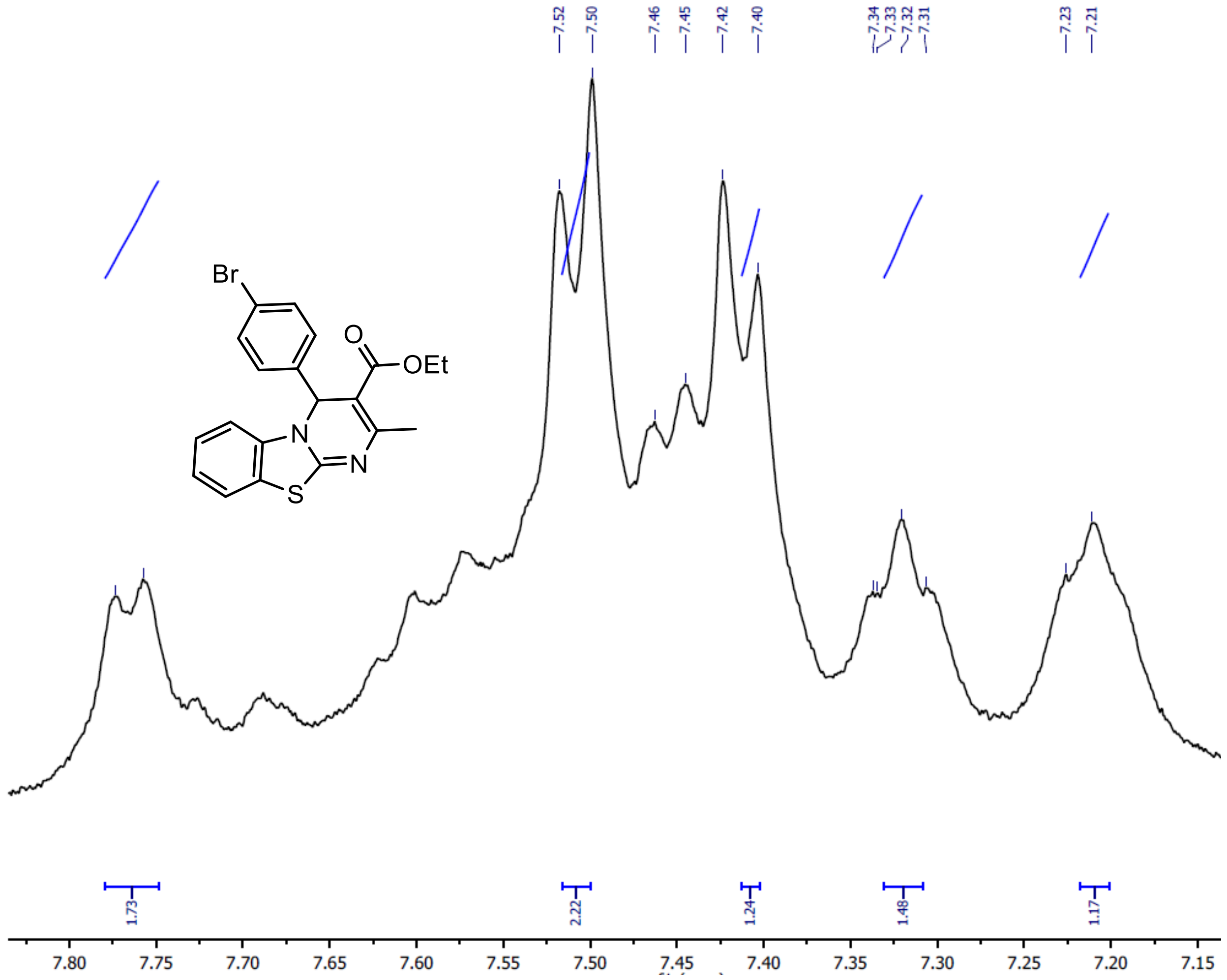

S14. The ^{1}H NMR (400MHz) spectrum of Ethyl-2-methyl-4-(4-bromo phenyl)-4*H*-pyrimido[2,1-*b*][1,3]benzothiazole-3-carboxylate

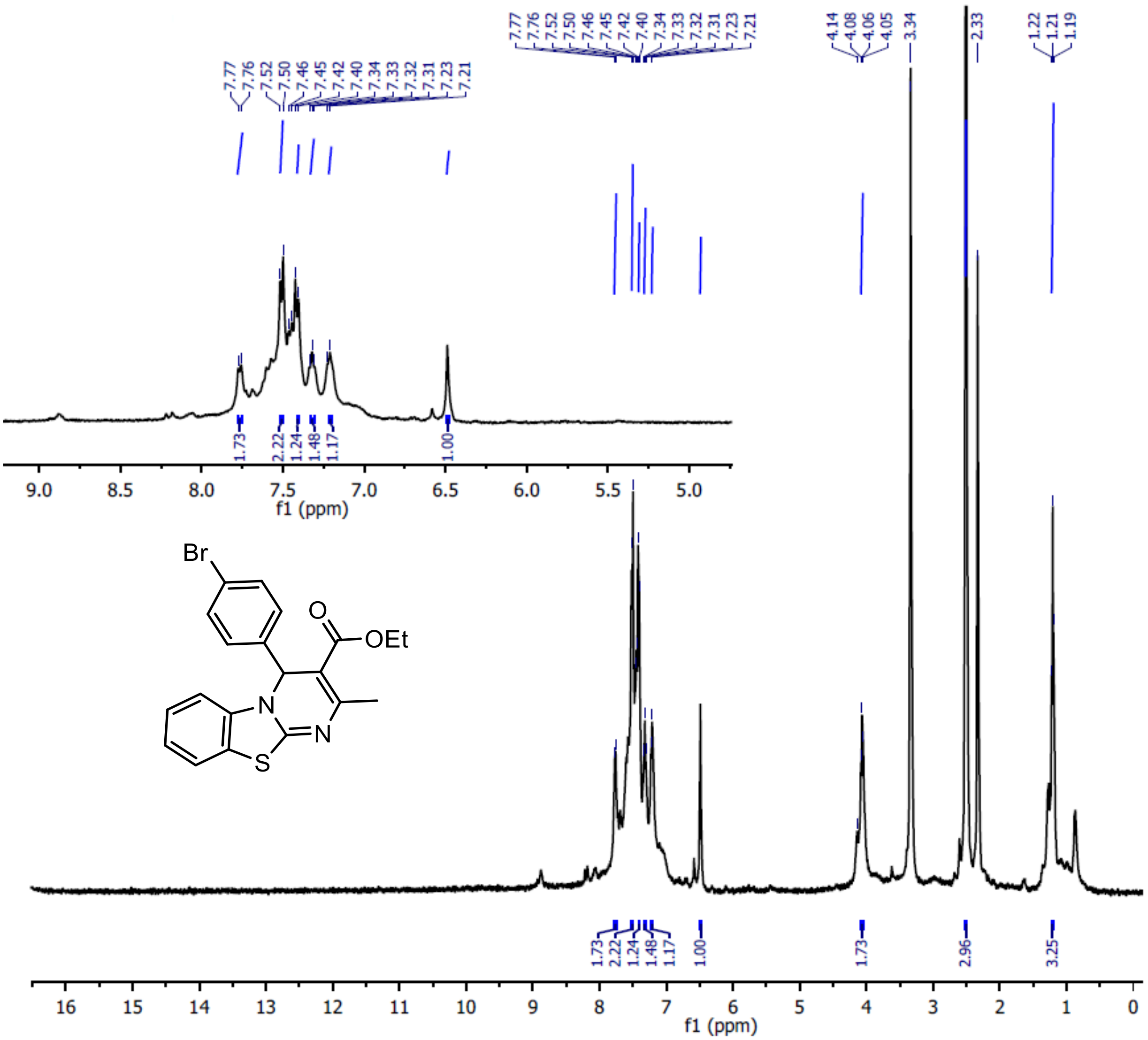

S15. The ^{1}H NMR (400MHz) spectrum of Ethyl-2-methyl-4-(4-bromo phenyl)-4*H*-pyrimido[2,1-*b*][1,3]benzothiazole-3-carboxylate

Ethyl-2-methyl-4-(4-hydroxy phenyl)-4*H*-pyrimido[2,1-*b*][1,3]benzothiazole-3-carboxylate

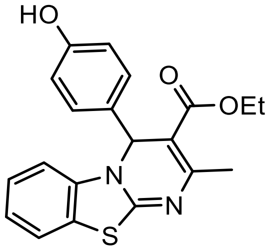

Pale yellow solid. ^{1}H NMR (DMSO-d$_6$, 400 MHz): δ 9.50 (s, 1H), 7.71 (d, *J*=6.2 Hz, 1H), 7.18-7.41 (m, 5H), 6.65 (d, *J*=7.7 Hz 2H), 6.33 (s, 1H), 4.03-4.14 (m, 2H), 2.31 (s, 3H), 1.19 (s, 3H). IR (KBr): 2976, 1675, 1593, 1511, 1451, 1363, 1327, 1269, 1238, 1170, 1023, 839, 743 cm^{-1}. mp: 210-212 °C.

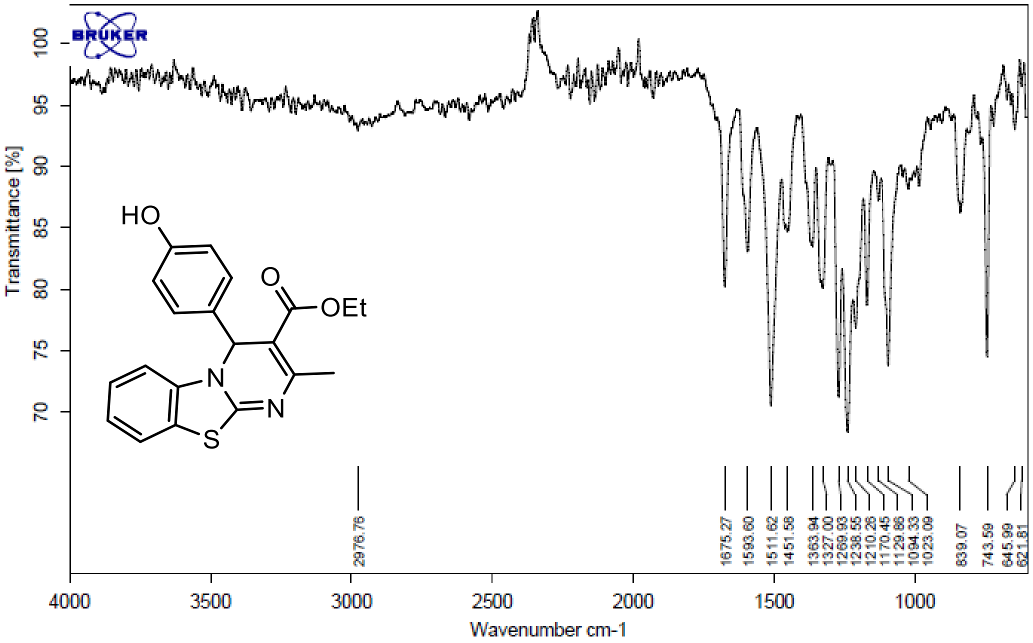

S16. The FT-IR spectrum of Ethyl-2-methyl-4-(4-hydroxy phenyl)-4*H*-pyrimido[2,1-*b*][1,3]benzothiazole-3-carboxylate

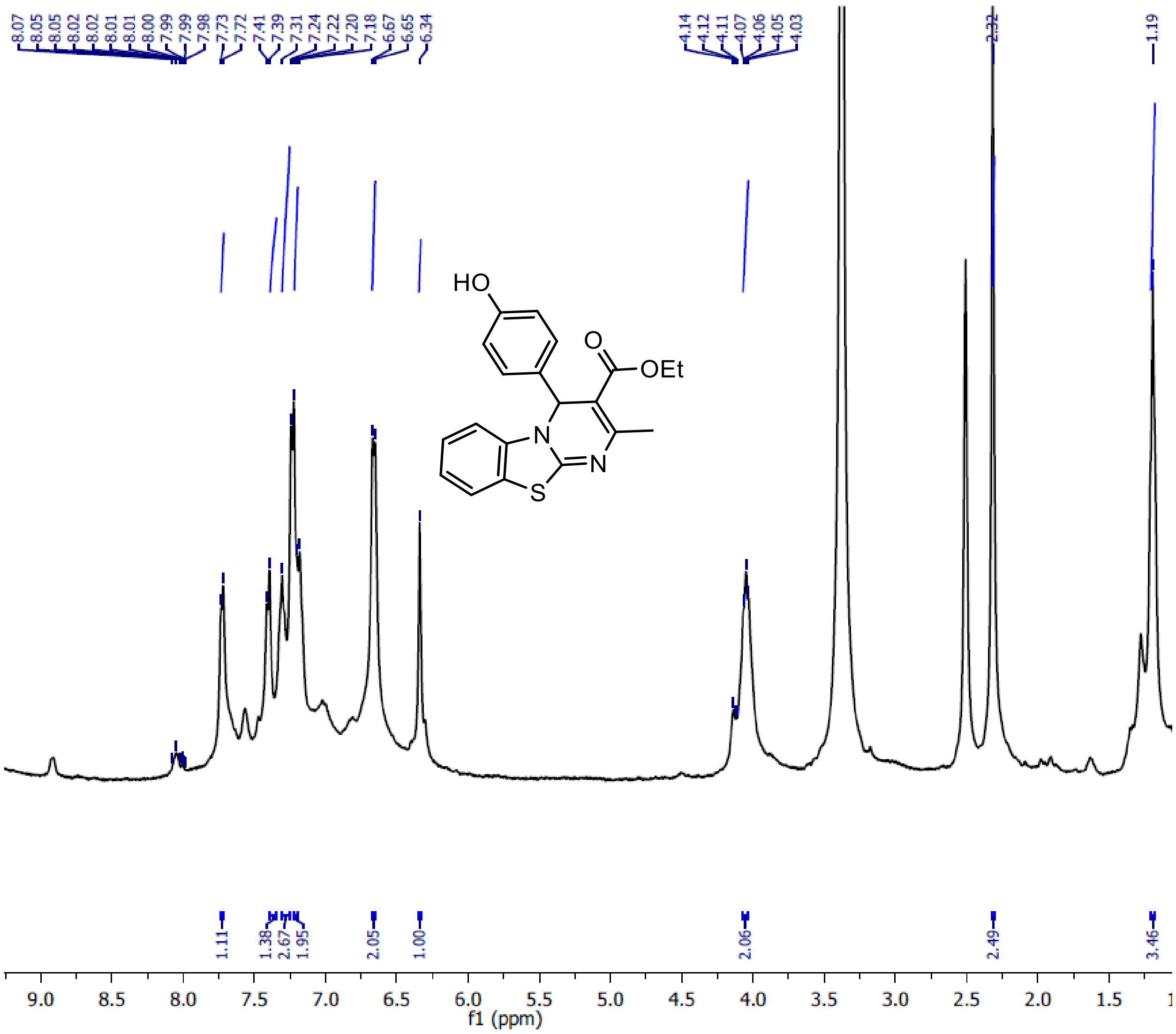

S17. The ^{1}H NMR (400MHz) spectrum of Ethyl-2-methyl-4-(4-hydroxy phenyl)-4*H*-pyrimido[2,1-*b*][1,3]benzothiazole-3-carboxylate

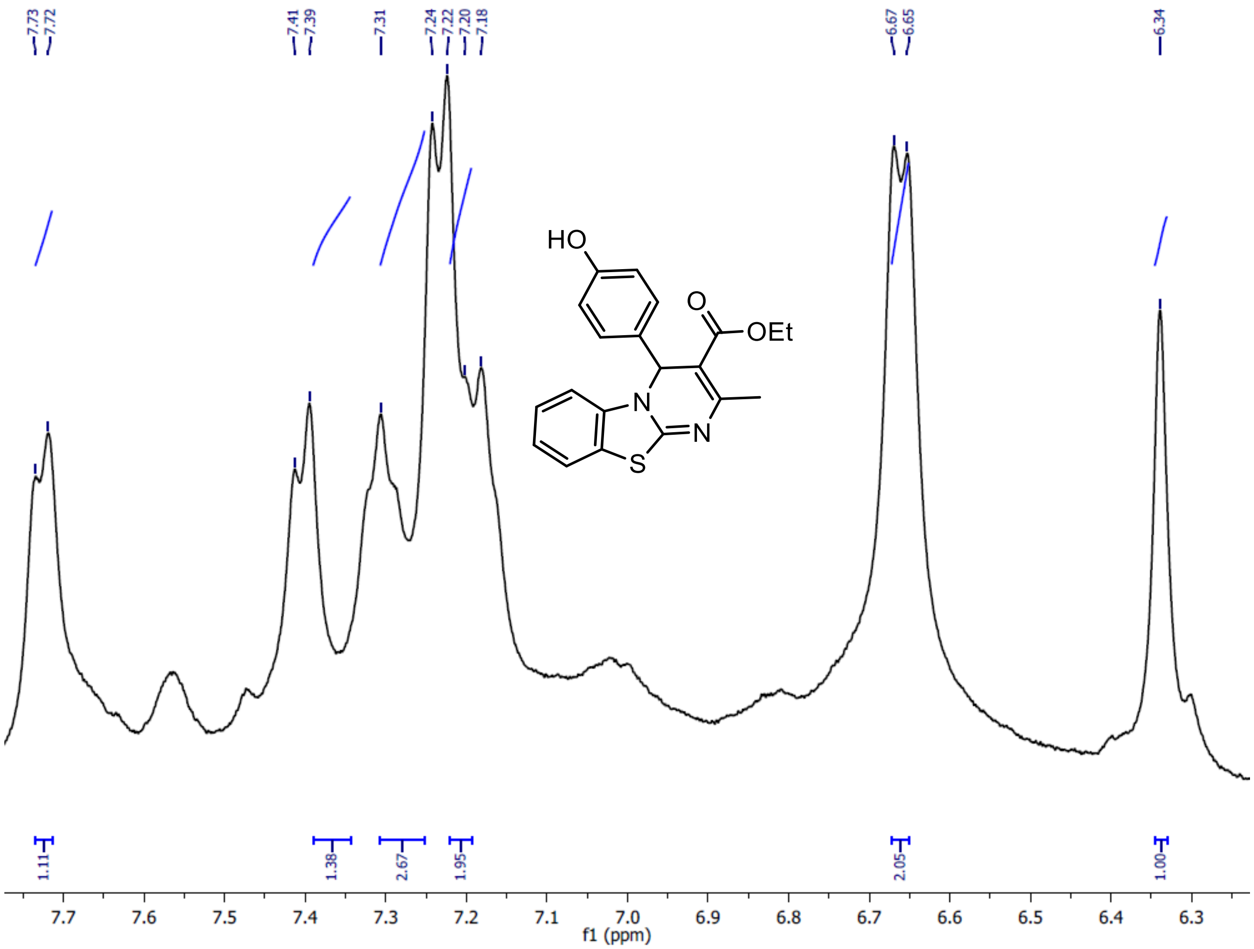

S18. The ^{1}H NMR (400MHz) spectrum of Ethyl-2-methyl-4-(4-hydroxy phenyl)-4*H*-pyrimido[2,1-*b*][1,3]benzothiazole-3-carboxylate

Ethyl-2-methyl-4-(2-nitrophenyl)-4*H*-pyrimido[2,1-*b*][1,3]benzothiazole-3-carboxylate

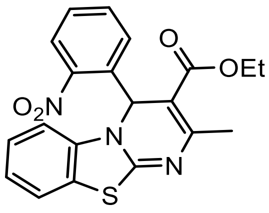

Red orange solid. ^{1}H NMR (DMSO-d$_6$, 400 MHz): δ 7.9 (d, *J* = 8 Hz, 1H) ,7.80 (d, *J*=7.7 Hz, 1H), 7.65-7.72 (m, 2H), 7.56 (d, *J*=7.5 Hz, 1H),7.48 (t, *J*=7.6 Hz, 1H) 7.33 (q, 1H), 7.24 (t, *J*= 8 Hz, 1H), 7.19 (s, 1H), 4.03-4.08 (m, 2H), 2.51 (s, 3H), 1.11 (t, *J*=6.7 Hz, 3H). IR (KBr): 2983, 1665, 1584, 1507, 1443, 1360, 1328, 1272, 1241, 1201, 1096, 741 cm^{-1}. mp: 122-124 °C.

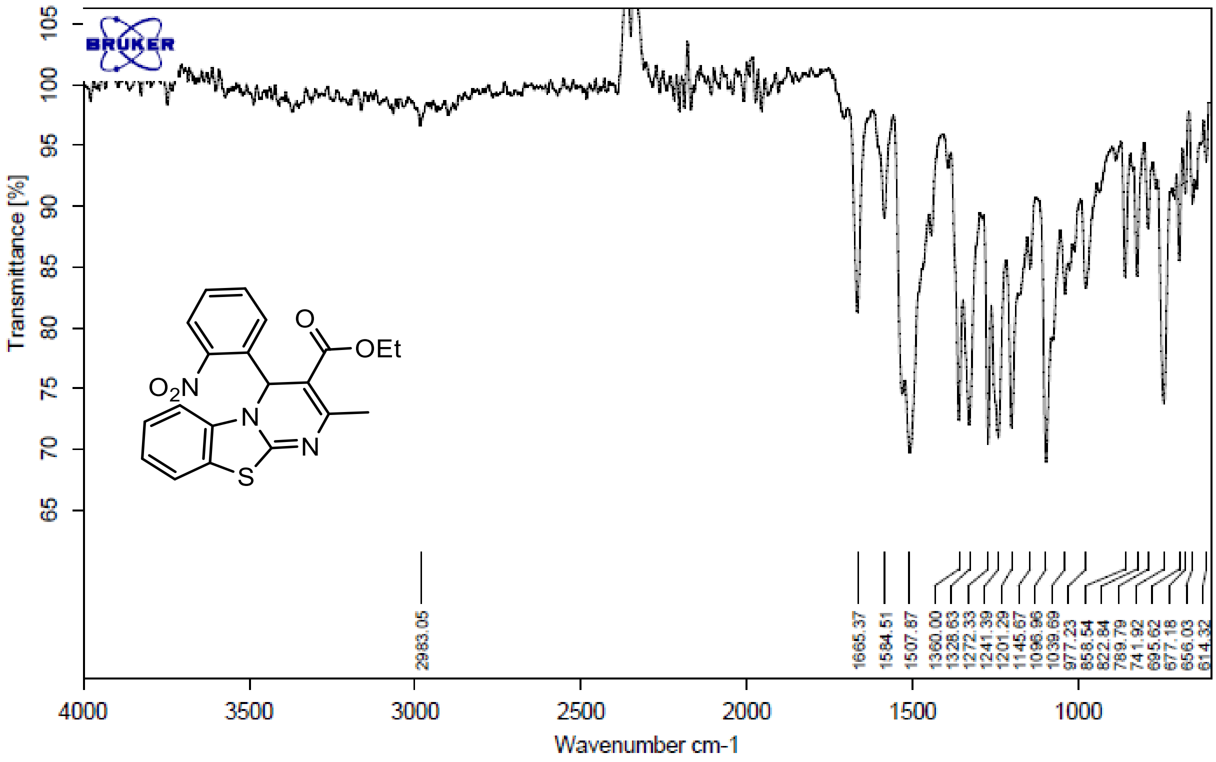

S19. The FT-IR spectrum of Ethyl-2-methyl-4-(2-nitrophenyl)-4*H*-pyrimido[2,1-*b*][1,3]benzothiazole-3-carboxylate

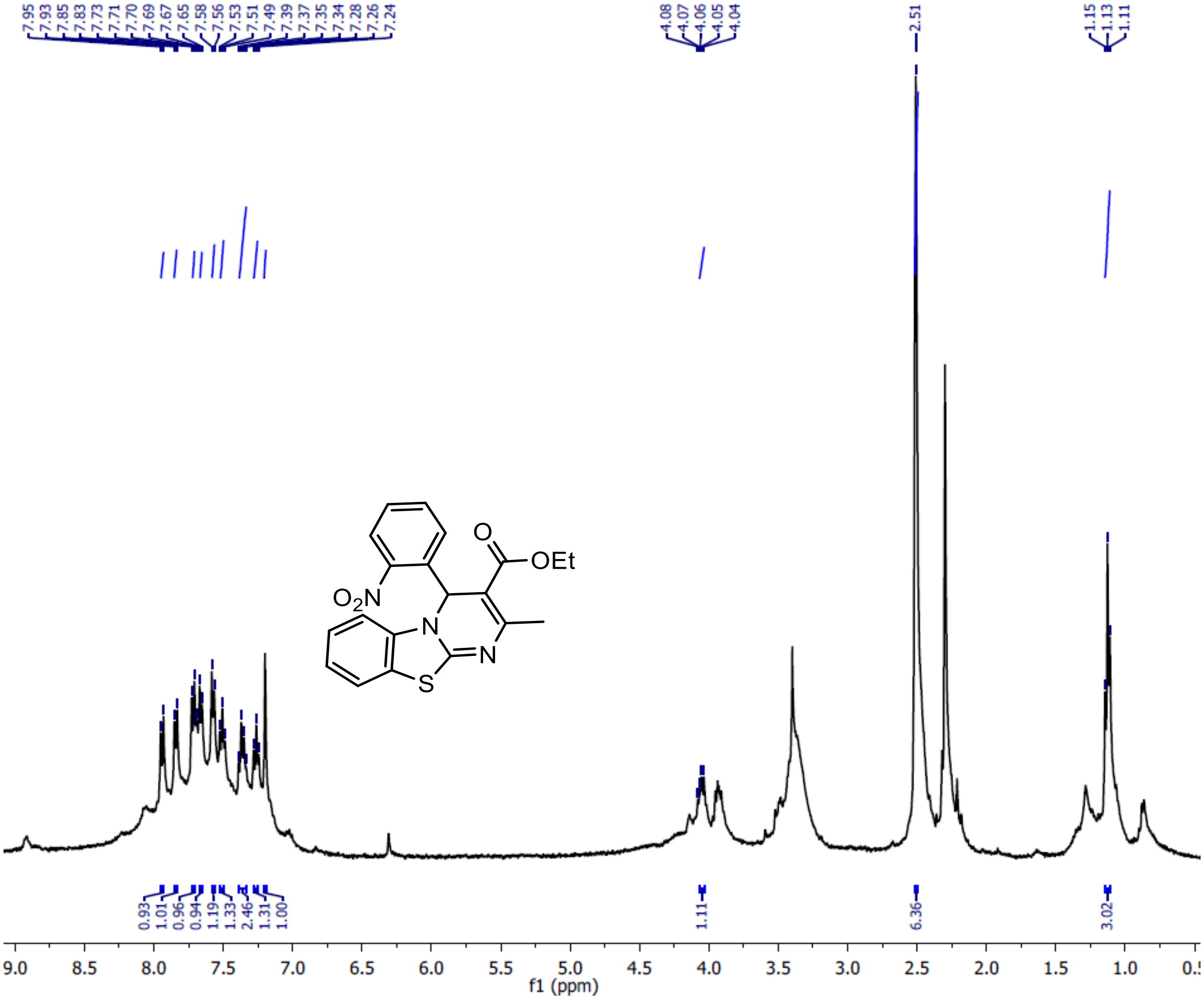

S20. The ^{1}H NMR (400MHz) spectrum of Ethyl-2-methyl-4-(2-nitrophenyl)-4*H*-pyrimido[2,1-*b*][1,3]benzothiazole-3-carboxylate

The ^{1}H NMR (400MHz) spectrum of Ethyl-2-methyl-4-(2-nitrophenyl)-4*H*-pyrimido[2,1-*b*][1,3]benzothiazole-3-carboxylate

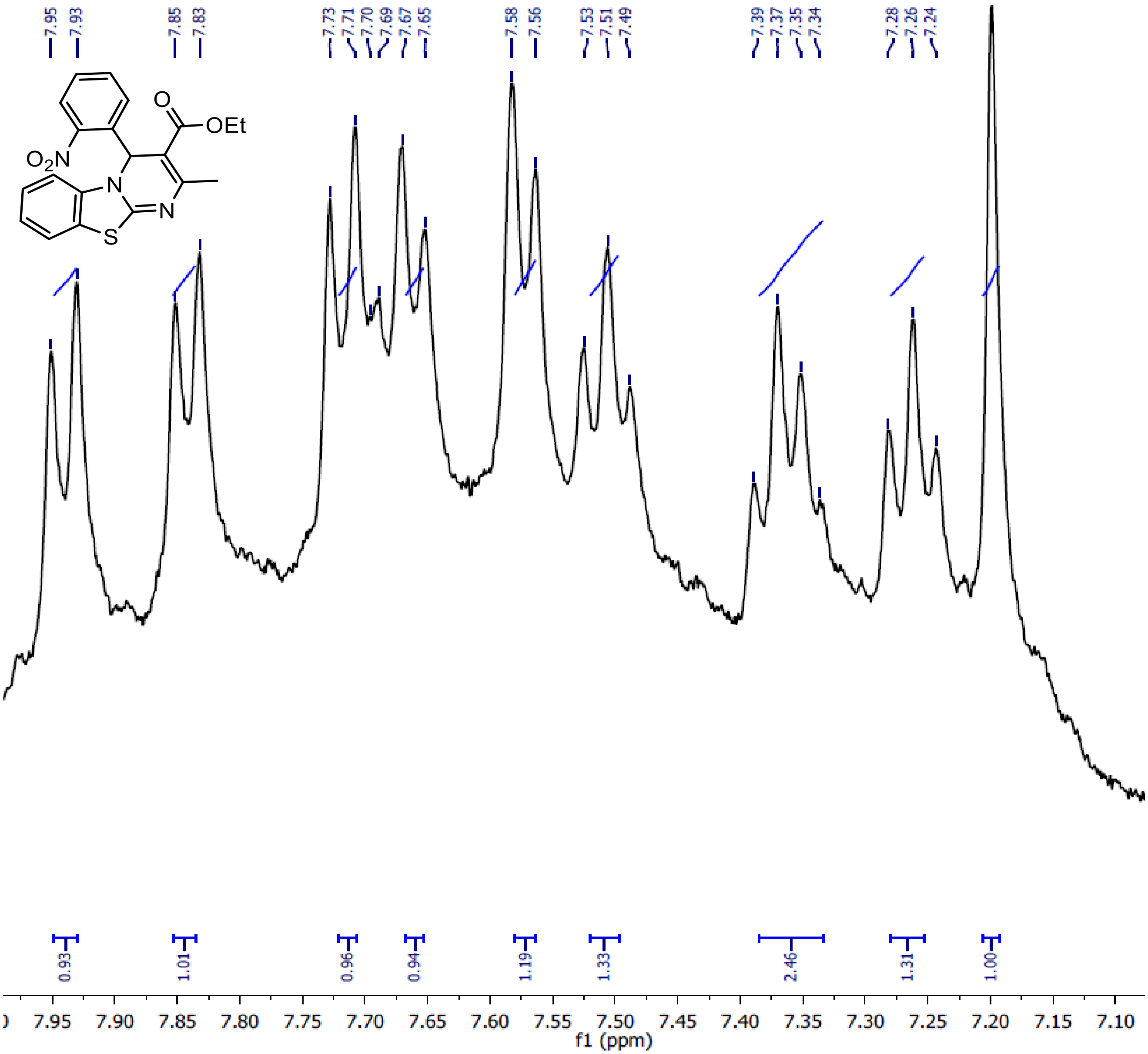

S21. The ^{1}H NMR (400MHz) spectrum of Ethyl-2-methyl-4-(2-nitrophenyl)-4*H*-pyrimido[2,1-*b*][1,3]benzothiazole-3-carboxylate

Ethyl-2-methyl-4-(2-chlorophenyl)-4*H*-pyrimido[2,1-*b*][1,3]benzothiazole-3-carboxylate

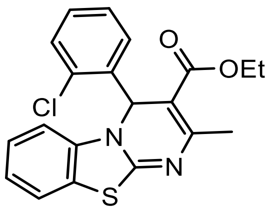

Yellow solid. ^{1}H NMR (DMSO-d$_6$, 400 MHz): δ 7.94 (d, *J*=8 Hz, 1H), 7.84 (d, *J*=8 Hz, 1H), 7.74 (d, *J*=8 Hz, 1H), 7.67 (m, 1H), 7.49-7.54 (m, 2H), 7.38 (t, *J*=7.2 Hz, 1H), 7.25-7.31 (m, 2H), 3.97-3.99 (m, 1H), 4.01-4.15 (m, 1H), 2.32 (s, 1H), 1.14 (t, 3H). IR (KBr): 2969, 1674, 1592, 1473, 1367, 1325, 1268, 1237, 1095, 746 cm^{-1}. mp: 122-123 °C.

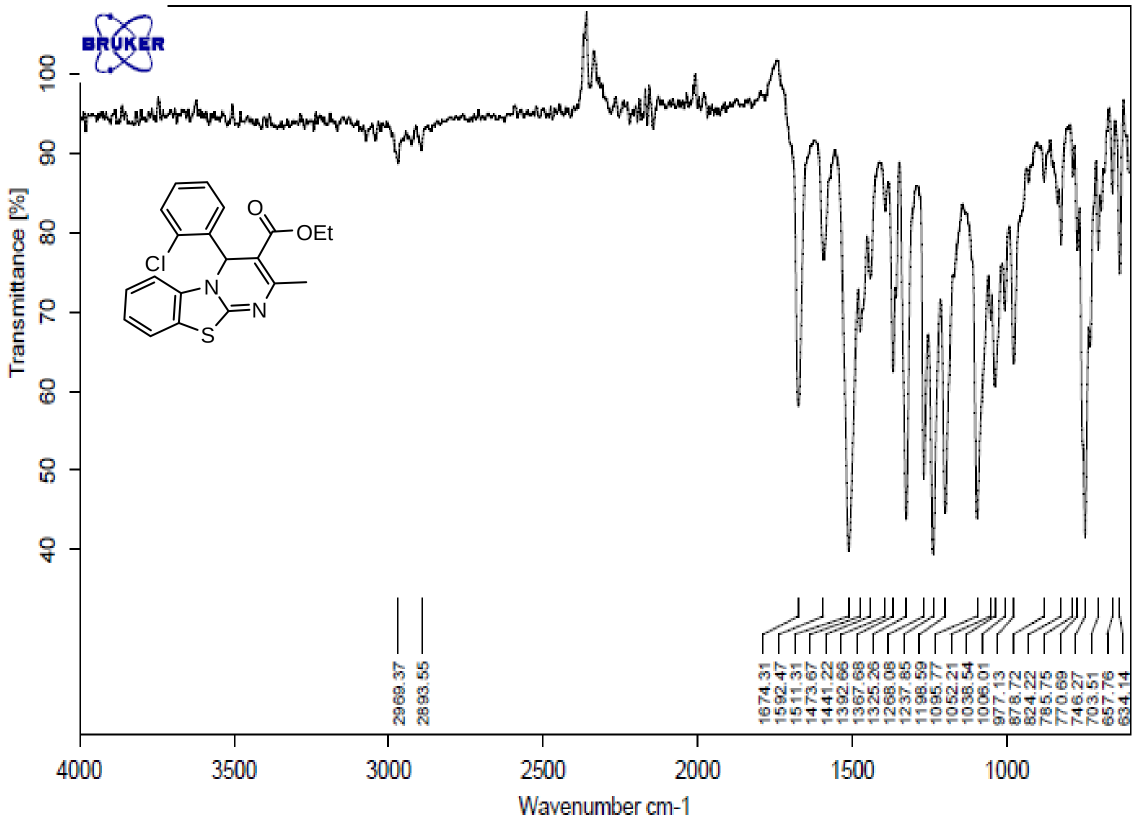

S22. The FT-IR spectrum of Ethyl-2-methyl-4-(2-chlorophenyl)-4*H*-pyrimido[2,1-*b*][1,3]benzothiazole-3-carboxylate

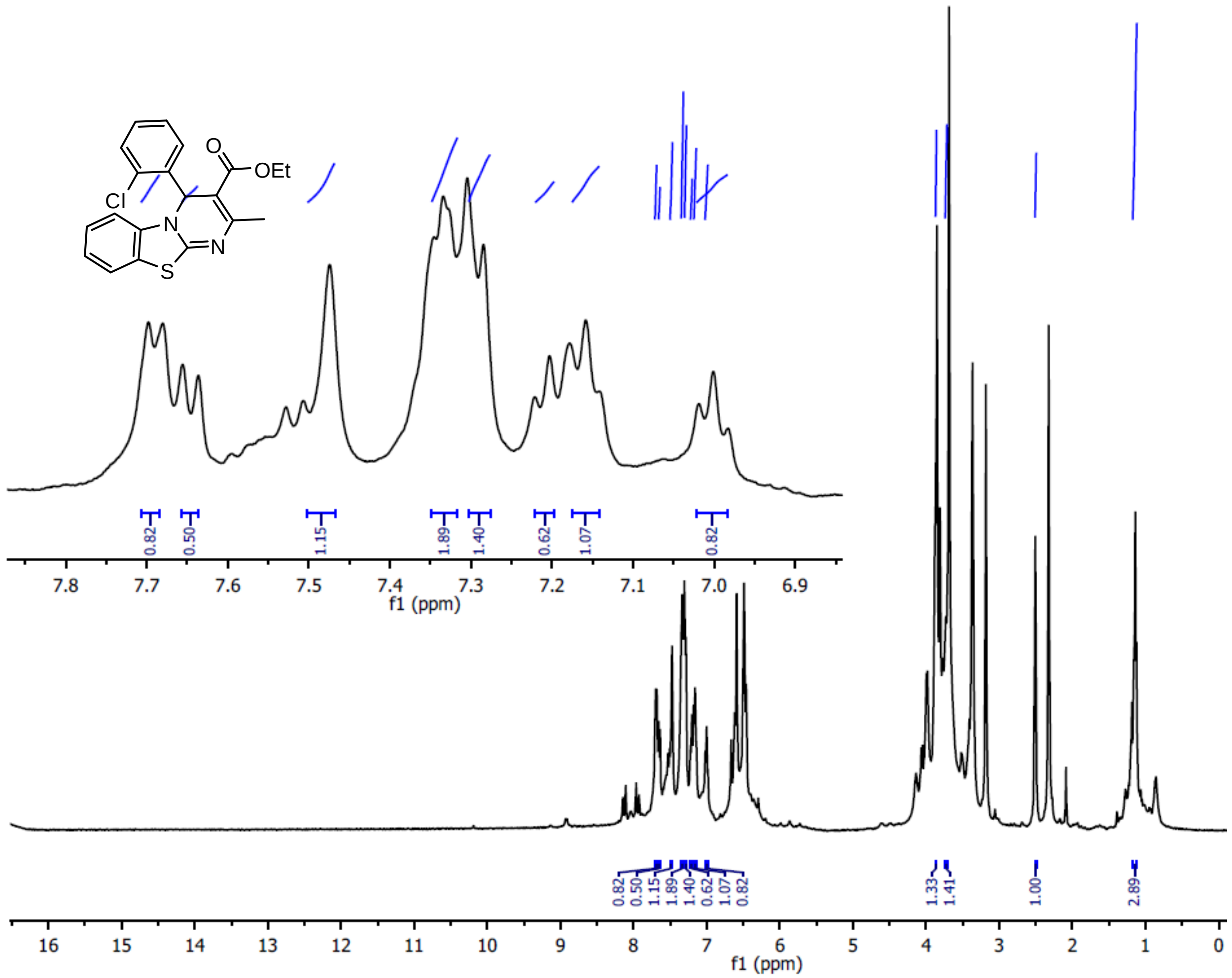

S23. The ^{1}H NMR (400MHz) spectrum of Ethyl-2-methyl-4-(2-chlorophenyl)-4*H*-pyrimido[2,1-*b*][1,3]benzothiazole-3-carboxylate

Ethyl-2-methyl-4-(2-ethoxy phenyl)-4*H*-pyrimido[2,1-*b*][1,3]benzothiazole-3-carboxylate

Yellow solid. ^{1}H NMR (DMSO-d$_6$, 400 MHz): δ 7.72 (d, *J*=7.4 Hz, 1H), 7.54 (d, *J*=8 Hz, 1H), 7.22-744 (m, 4H), 6.82 (d, *J*=8 Hz, 2H), 6.40 (s, 1H), 4.11-4.13 (m, 2H), 4.03 (t, *J*=7 Hz, 2H), 2.32 (s, 3H), 1.24 (t, *J*=6.8 Hz, 3H), 1.18 (t, *J*=7 Hz 3H). IR (KBr): 2980, 1692, 1600, 1511, 1493, 1242, 1201, 1075, 1040, 752 cm^{-1}. mp: 171-173 $^{\circ}$C.

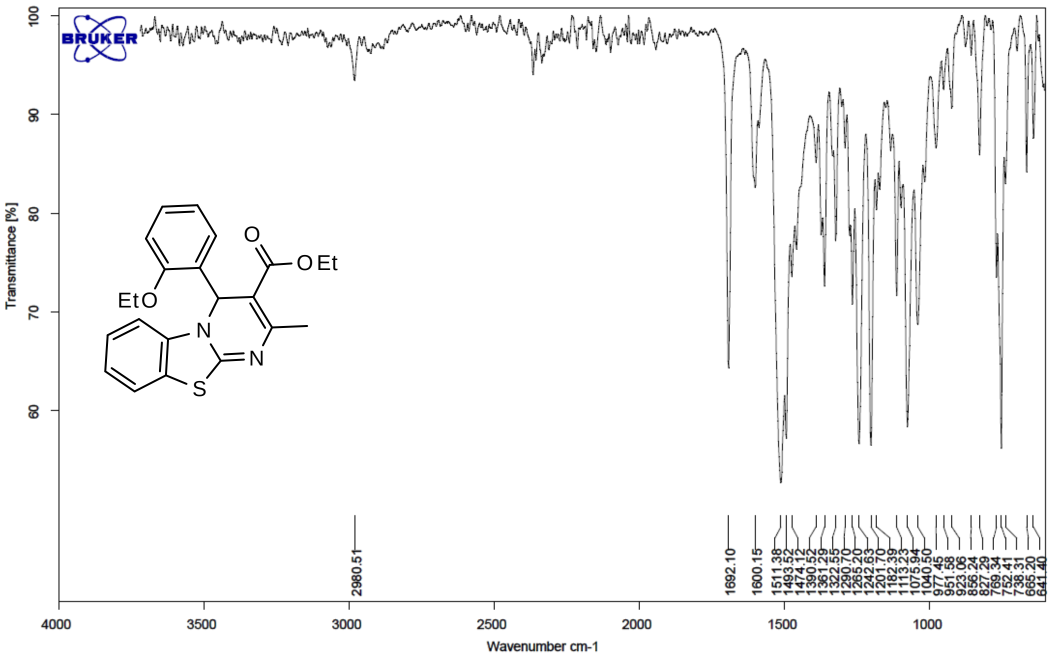

S24. The FT-IR spectrum of Ethyl-2-methyl-4-(2-ethoxy phenyl)-4*H*-pyrimido[2,1-*b*][1,3]benzothiazole-3-carboxylate

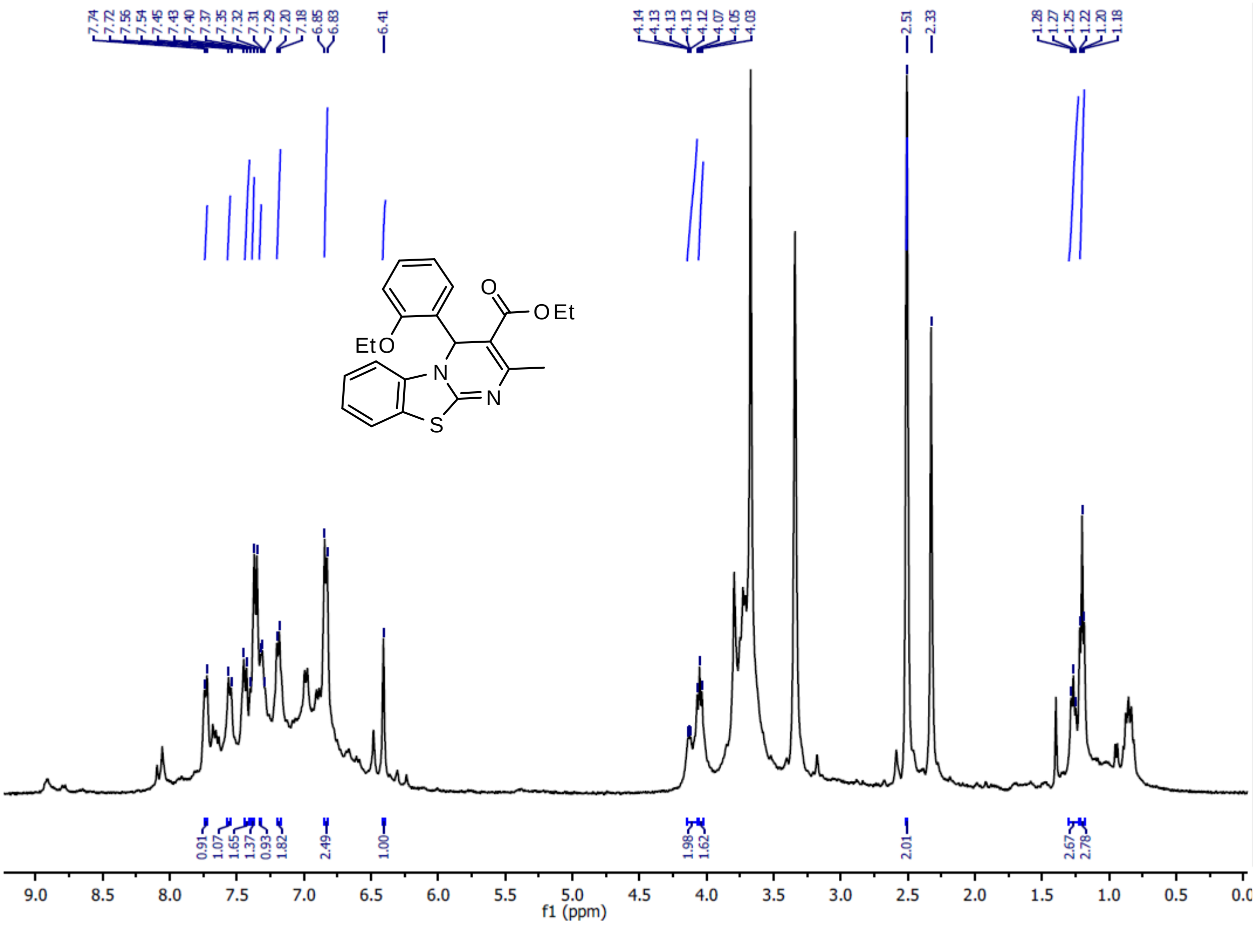

S25. The ^{1}H NMR (400MHz) spectrum of Ethyl-2-methyl-4-(2-ethoxy phenyl)-4*H*-pyrimido[2,1-*b*][1,3]benzothiazole-3-carboxylate

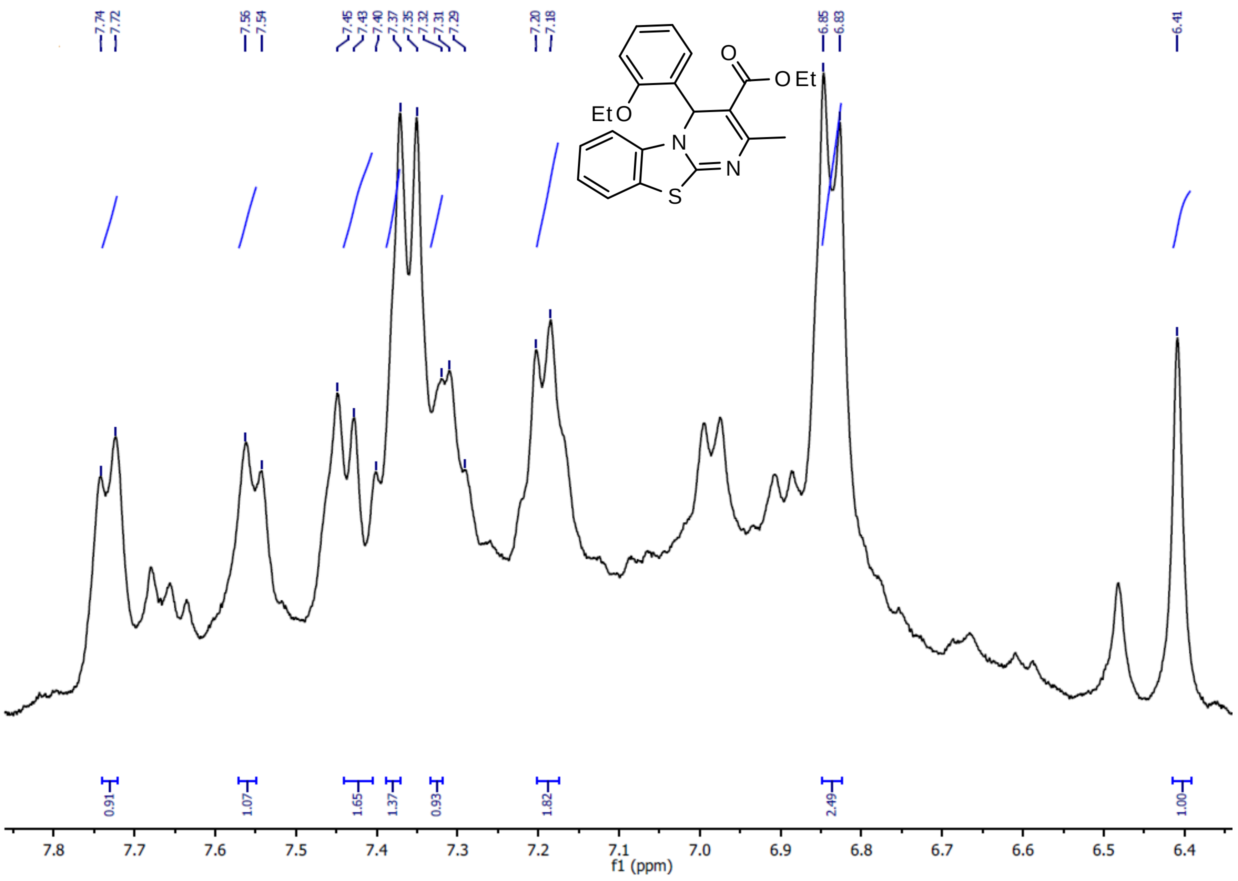

S26. The ¹H NMR (400MHz) spectrum of Ethyl-2-methyl-4-(2-ethoxy phenyl)-4*H*-pyrimido[2,1-*b*][1,3]benzothiazole-3-carboxylate

Ethyl-2-methyl-4-(3-nitrophenyl)-4*H*-pyrimido[2,1-*b*][1,3]benzothiazole-3-carboxylate

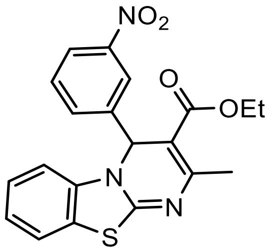

Light yellow solid. ¹H NMR (DMSO-d6, 400 MHz): δ 8.36 (s, 1H), 8.09 (m, 1H), 7.91 (m, 1H), 7.86 (m, 1H), 7.77 (m, 1H), 7.55 (m, 1H), 7.31 (m, 1H), 7.21 (m, 1H), 6.71 (s, 1H), 4.03-4.14

(m, 2H), 2.35 (s, 3H), 1.22 (m, 3H). IR (KBr): 1654, 1581, 1502, 1334, 1272, 1248, 1204, 1097, 747 cm^{-1}. mp: 222-223 °C.

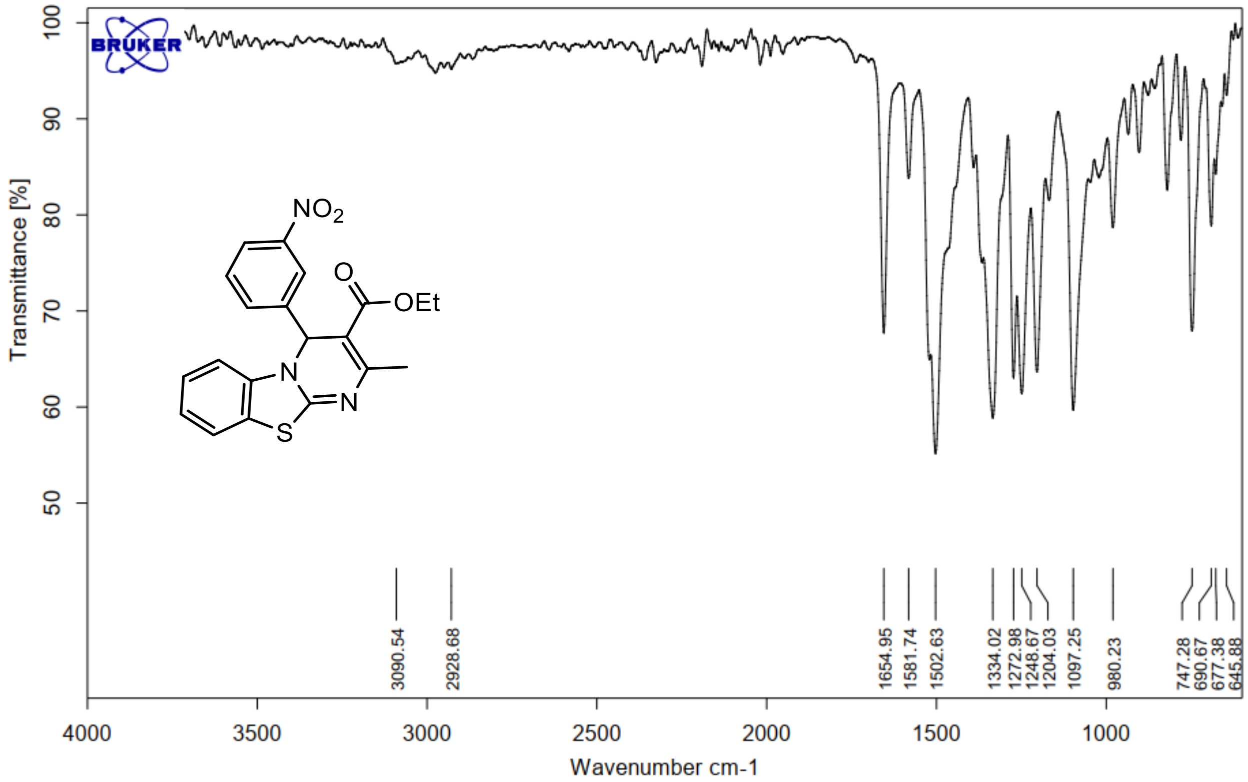

S27. The FT-IR spectrum of Ethyl-2-methyl-4-(3-nitrophenyl)-4*H*-pyrimido[2,1-*b*][1,3]benzothiazole-3-carboxylate

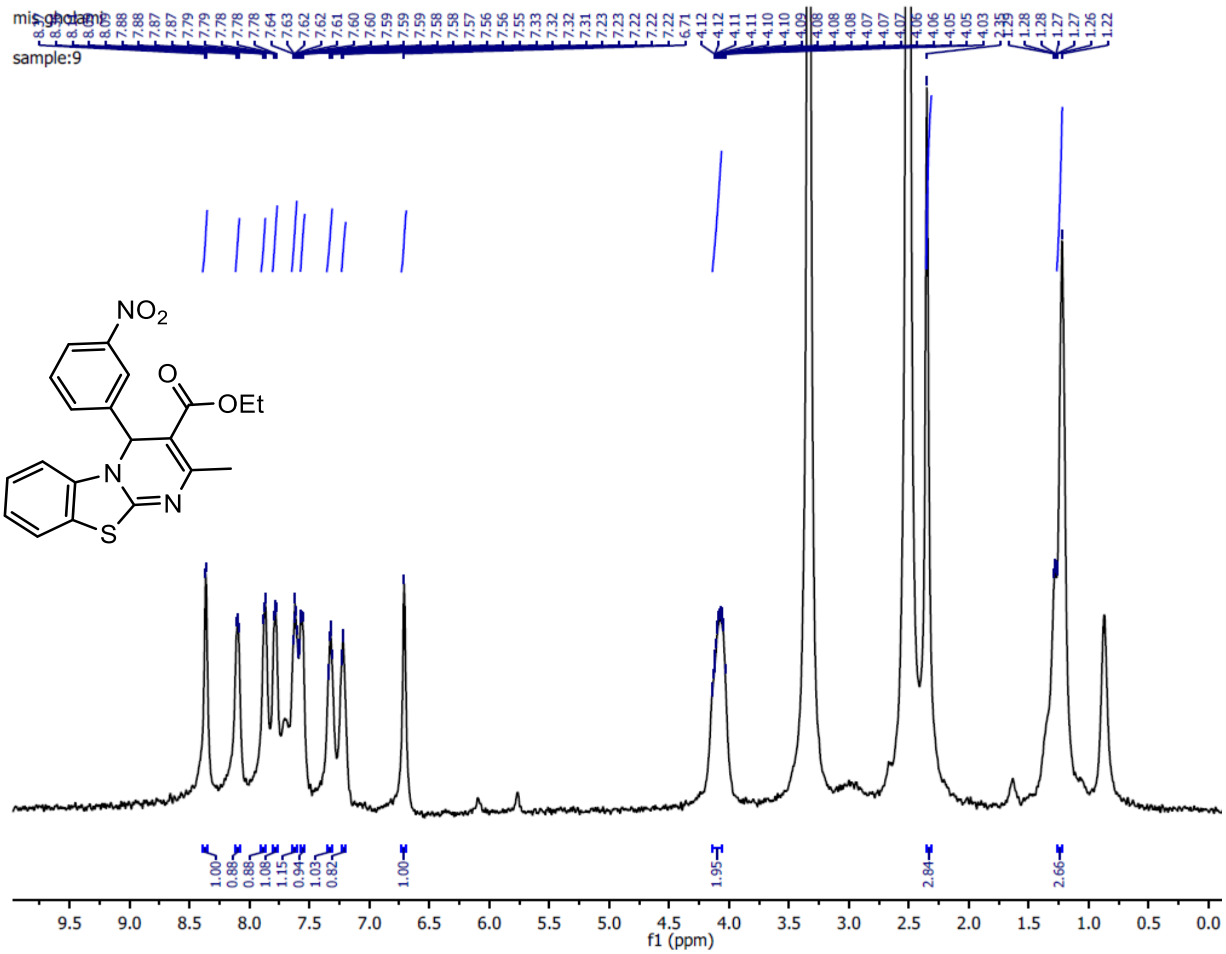

S28. The ^{1}H NMR (400MHz) spectrum of Ethyl-2-methyl-4-(3-nitrophenyl)-4*H*-pyrimido[2,1-*b*][1,3]benzothiazole-3-carboxylate

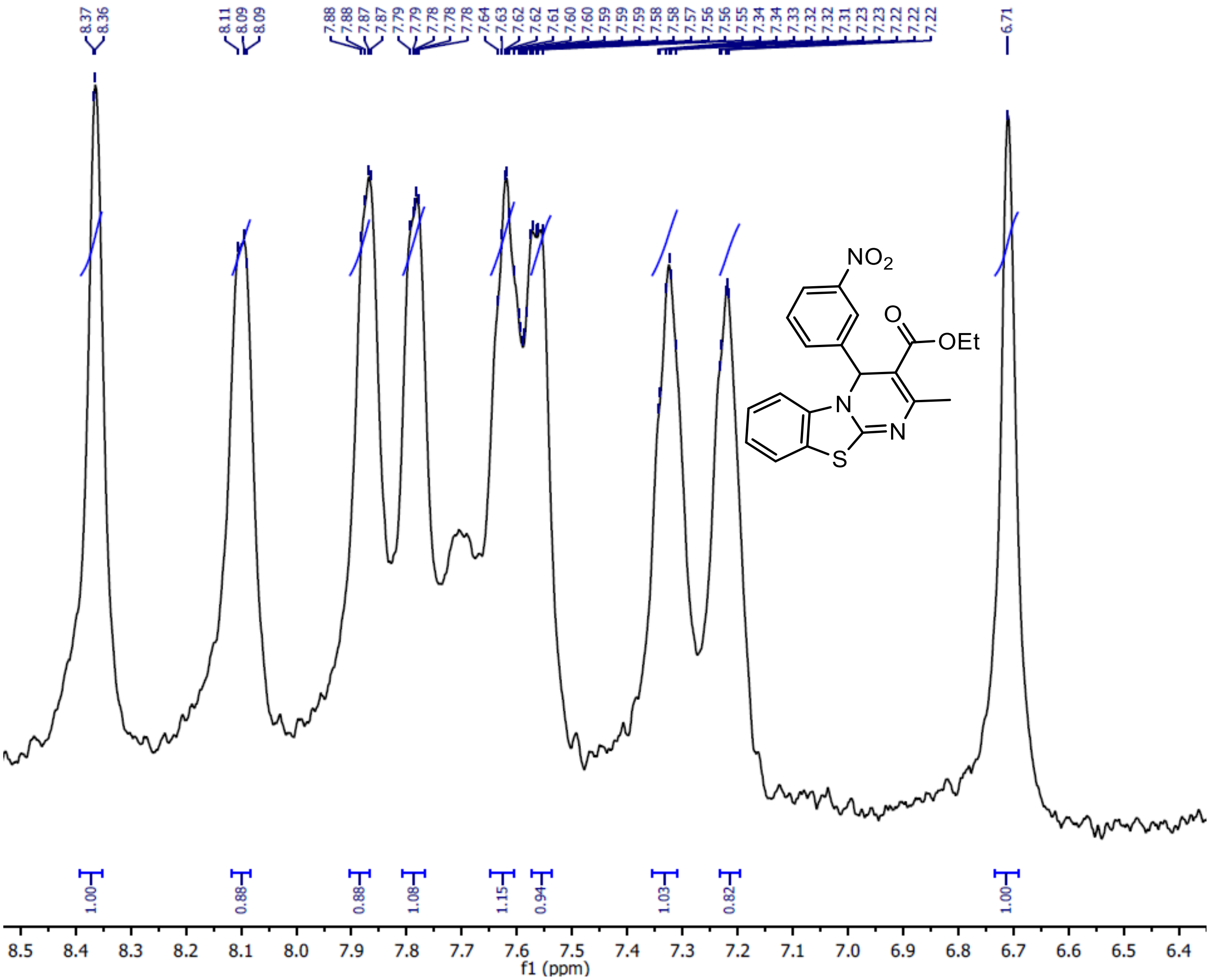

S29. The ^{1}H NMR (400MHz) spectrum of Ethyl-2-methyl-4-(3-nitrophenyl)-4*H*-pyrimido[2,1-*b*][1,3]benzothiazole-3-carboxylate

Ethyl-2-methyl-4-(3-hydroxy phenyl)-4*H*-pyrimido[2,1-*b*][1,3]benzothiazole-3-carboxylate

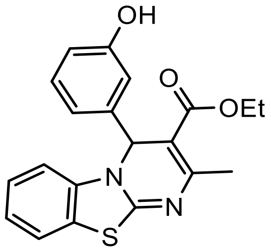

Yellow solid. ^{1}H NMR (DMSO-d_6, 400 MHz): δ 9.4 (s, 1H), 7.75 (s, 1H), 7.35 (m, 2H), 7.32 (m, 1H), 7.19 (s, 1H), 7.0 (s, 2H),6.80 (m, 1H), 6.61 (s, 1H), 6.38 (s, 1H), 4.07 (m, 2H), 2.31 (s, 3H), 1.21 (br. s, 3H). IR (KBr): 3062, 2981, 1683, 1595, 1507, 1456, 1274, 1241, 1212, 1098, 983, 785, 753, 740 cm^{-1}. mp: 261-262 °C.

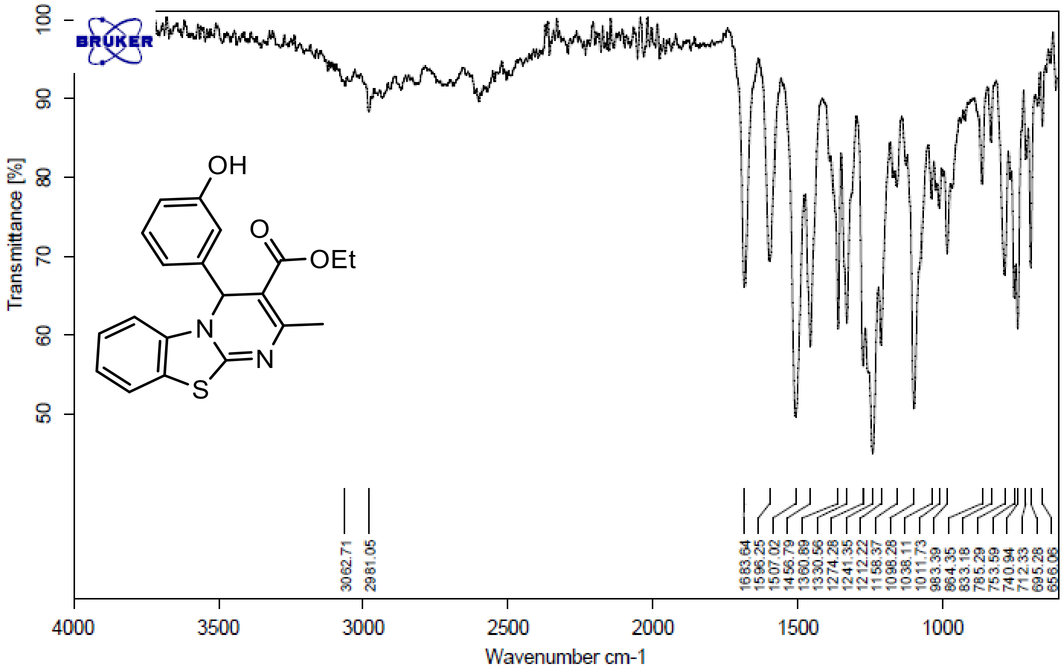

S30. The FT-IR spectrum of Ethyl-2-methyl-4-(3-hydroxy phenyl)-4*H*-pyrimido[2,1-*b*][1,3]benzothiazole-3-carboxylate

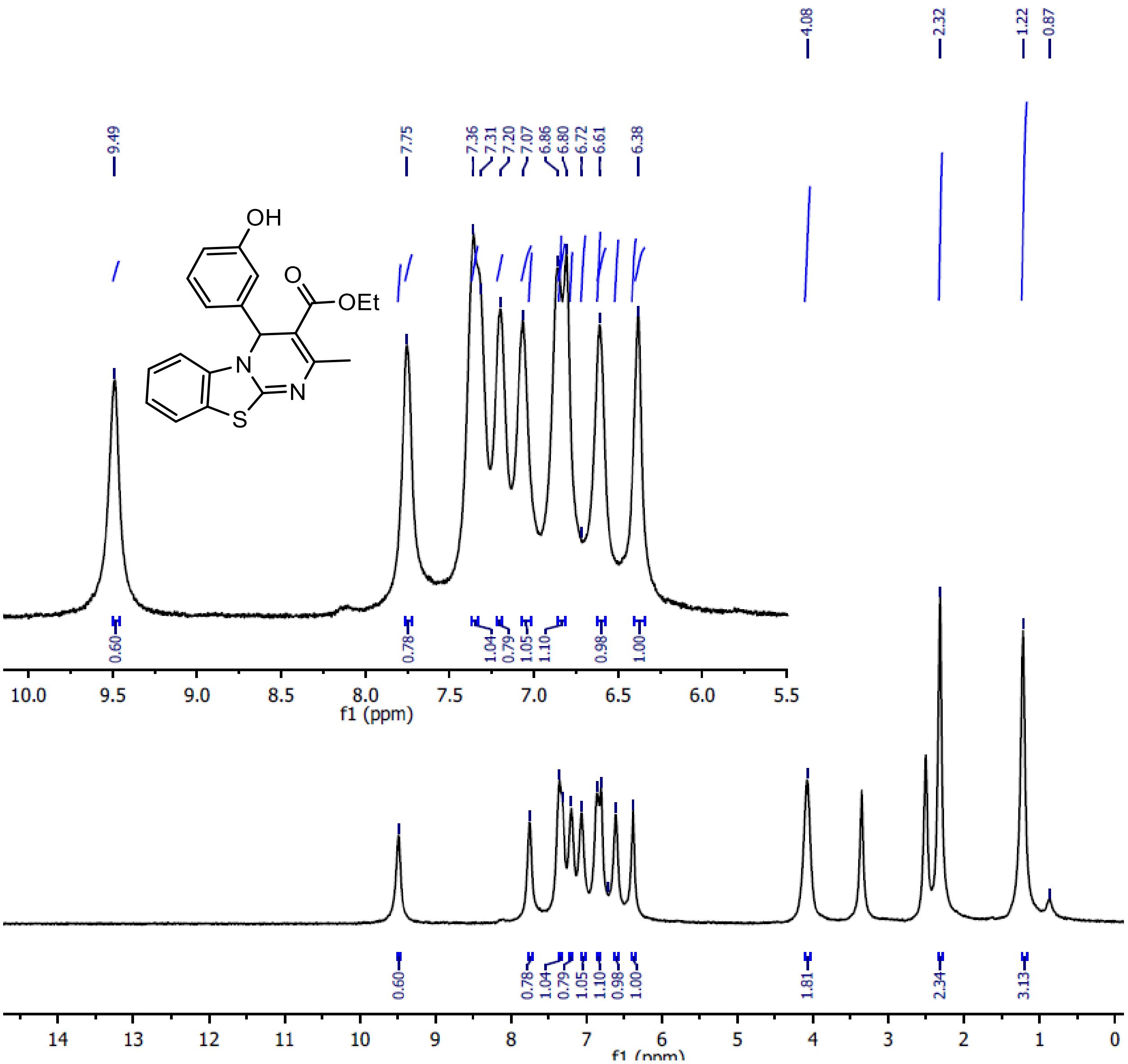

S31. The ^{1}H NMR (400MHz) spectrum of Ethyl-2-methyl-4-(3-hydroxy phenyl)-4*H*-pyrimido[2,1-*b*][1,3]benzothiazole-3-carboxylate

Ethyl-2-methyl-4-(2,4-dichlorophenyl)-4*H*-pyrimido[2,1-*b*][1,3]benzothiazole-3-carboxylate

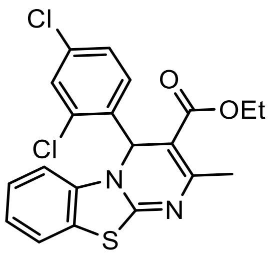

Yellow solid. ^{1}H NMR (DMSO-d$_6$, 400 MHz): δ 7.69-7.72 (m, 2H), 7.33-7.46 (m, 4H), 7.22 (m, , 1H), 6.77 (s, 1H), 4.04-4.06 (q, *J*= 6.8 Hz, 2H), 2.37 (s, 3H), 1.15 (t, *J*= 6.8 Hz, 3H). IR (KBr): 3007, 2971, 1698, 1583, 1490, 1360, 1242, 1201, 1076, 845, 743 cm^{-1}. mp: 133-134 °C.

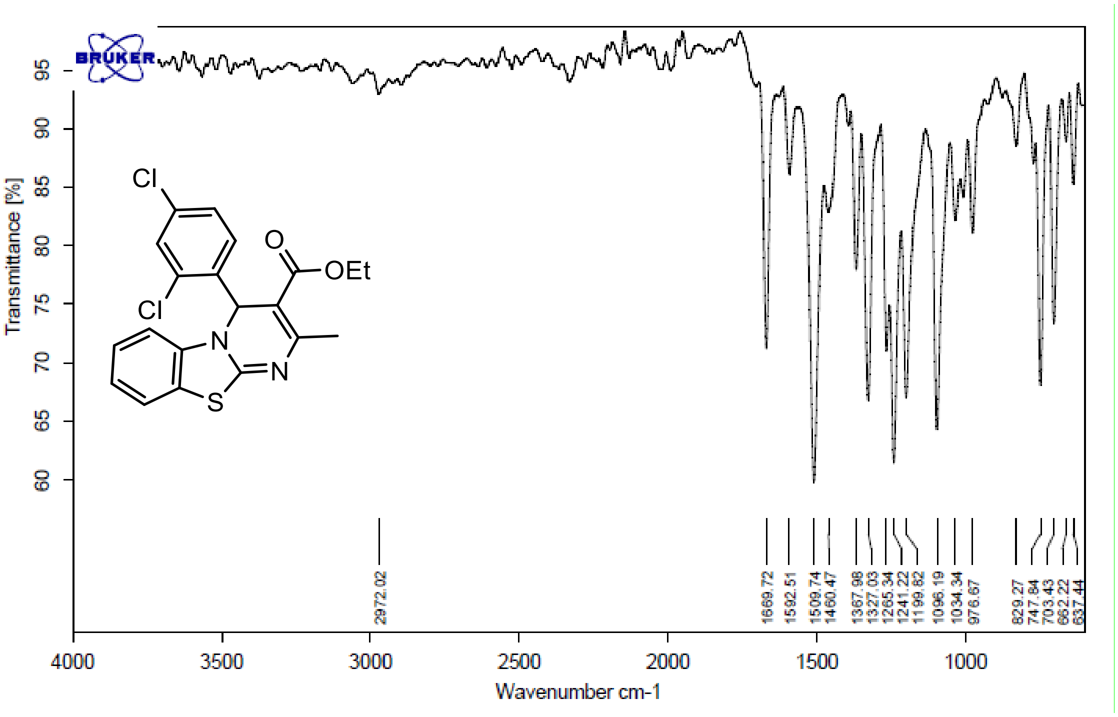

S32. The FT-IR spectrum of Ethyl-2-methyl-4-(2,4-dichlorophenyl)-4*H*-pyrimido[2,1-*b*][1,3]benzothiazole-3-carboxylate

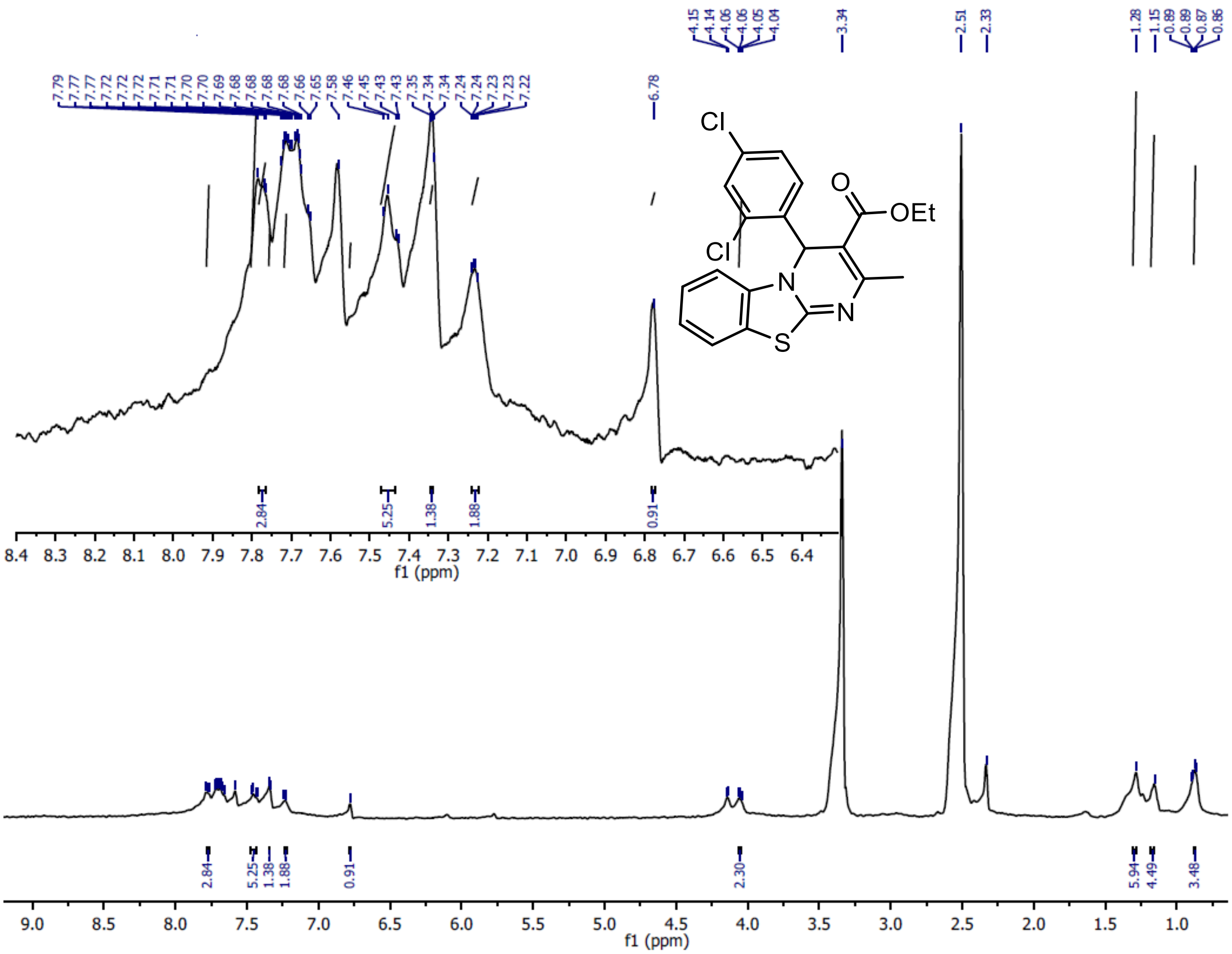

S33. The ^{1}H NMR (400MHz) spectrum of Ethyl-2-methyl-4-(2,4-dichlorophenyl)-4*H*-pyrimido[2,1-*b*][1,3]benzothiazole-3-carboxylate

Ethyl-2-methyl-4-(2,4-dimethoxyphenyl)-4*H*-pyrimido[2,1-*b*][1,3]benzothiazole-3-carboxylate

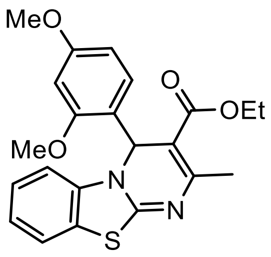

Yellow solid. ^{1}H NMR (DMSO-d_6, 400 MHz): δ 7.62 (d, *J*=7.6 Hz, 1H), 7.45 (d, *J*= 8 Hz, 1H), 7.37 (d, *J* = 8.4 Hz, 1H), 7.30 (td, *J* = 8 Hz, 1.2 Hz, 1H), 7.15 (td, *J* = 7.6 Hz, 1.2 Hz, 1H), 6.68

(s, 1H), 6.44-6.48 (m, 2H), 4.05 (q, J = 6.8 Hz, 2H), 3.93 (s, 3H), 3.71 (s, 3H), 2.36 (s, 3H), 1.19 (t, J = 6.8 Hz, 3H). IR (KBr): 2933, 2836, 1691, 1585, 1501, 1273, 1239, 1204, 1076, 822, 745 cm^{-1}. mp: 164-165 °C.

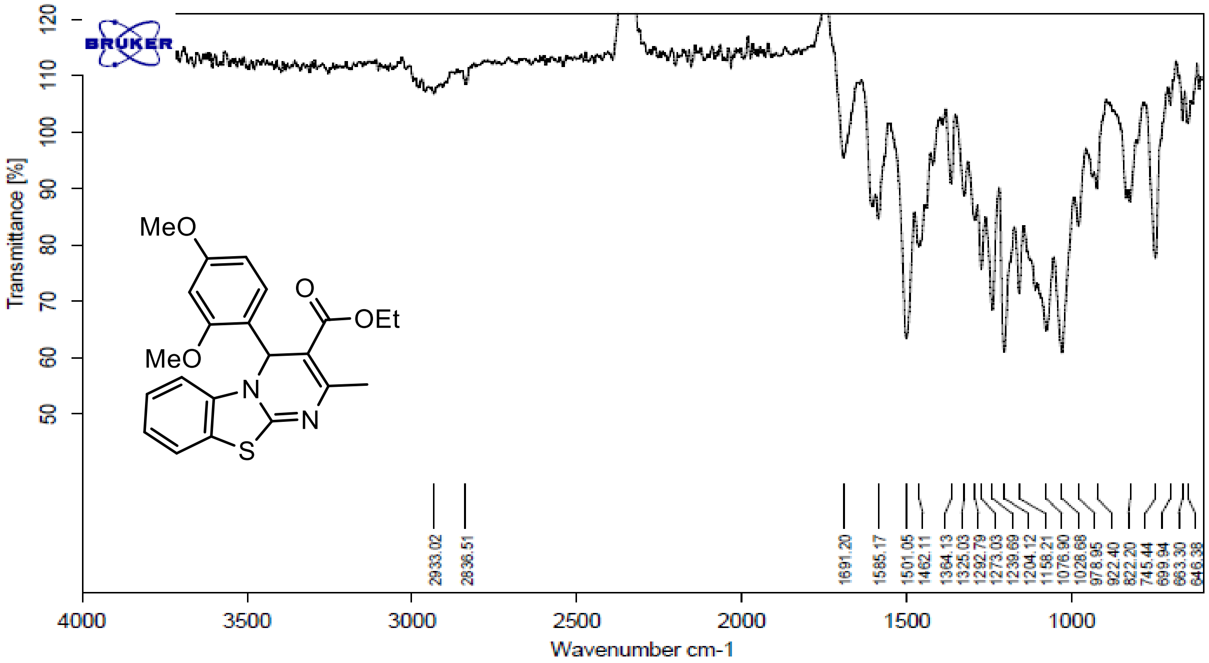

S34. The FT-IR spectrum of Ethyl-2-methyl-4-(2,4-dimethoxyphenyl)-4*H*-pyrimido[2,1-*b*][1,3]benzothiazole-3-carboxylate

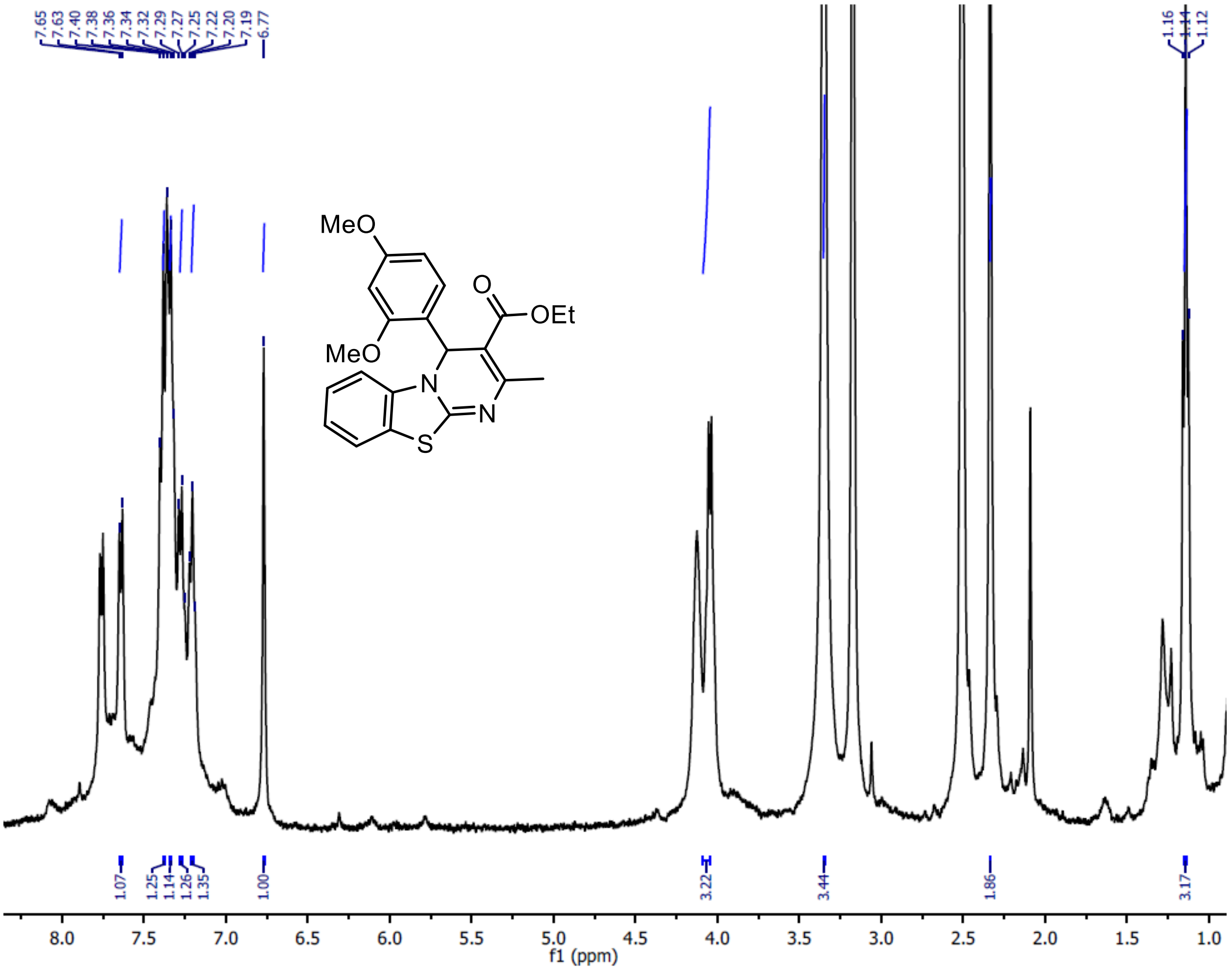

S35. The ^{1}H NMR (400MHz) spectrum of Ethyl-2-methyl-4-(2,4-dimethoxyphenyl)-4*H*-pyrimido[2,1-*b*][1,3]benzothiazole-3-carboxylate

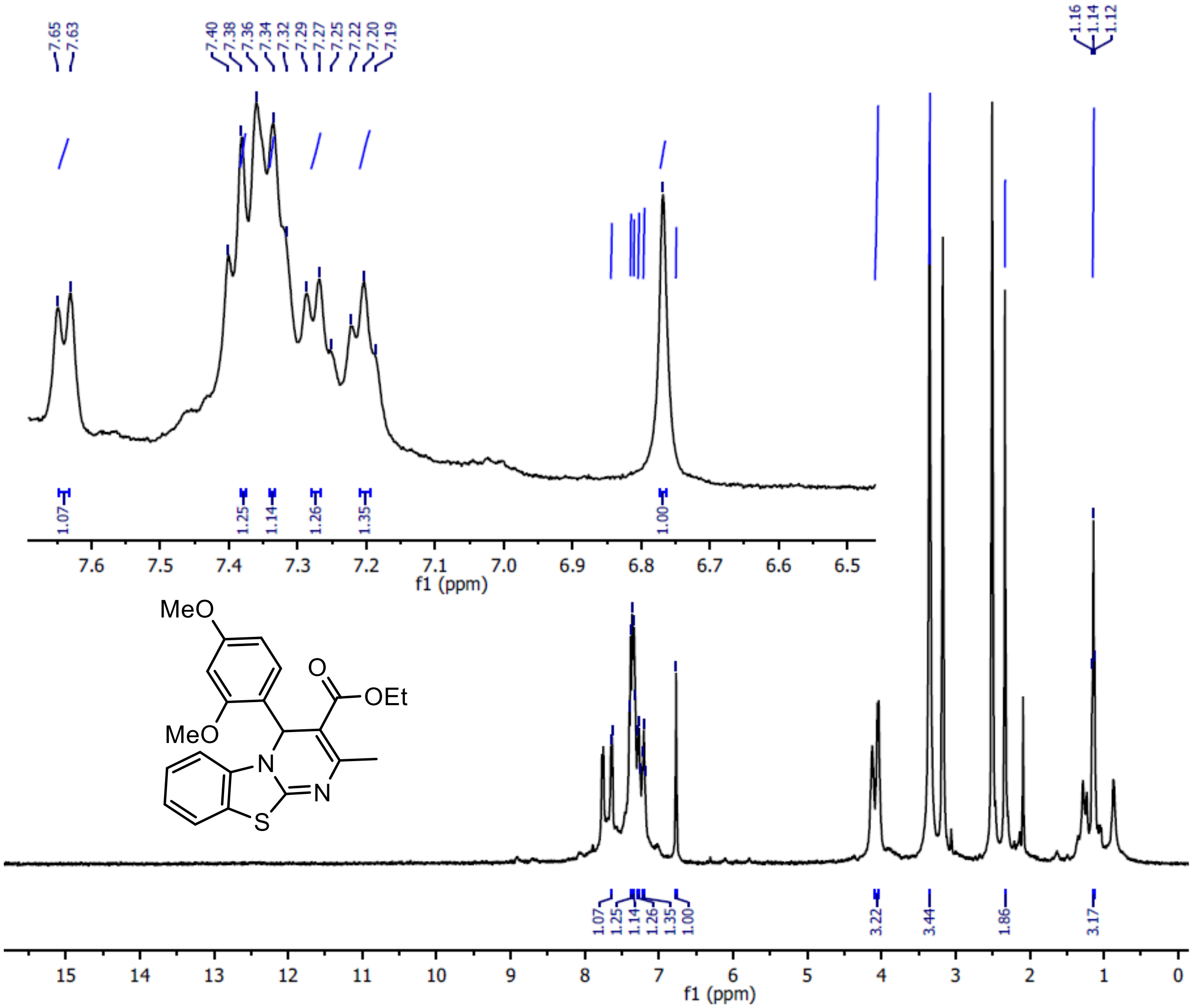

S36. The ^{1}H NMR (400MHz) spectrum of Ethyl-2-methyl-4-(2,4-dimethoxyphenyl)-4*H*-pyrimido[2,1-*b*][1,3]benzothiazole-3-carboxylate

Ethyl-2-methyl-4-(3,4-dihydroxyphenyl)-4*H*-pyrimido[2,1-*b*][1,3]benzothiazole-3-carboxylate

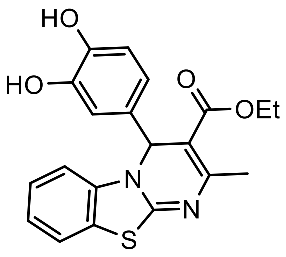

Gray solid. ^{1}H NMR (DMSO-d_6, 400 MHz): δ 8.30 (s, 1H), 7.91 (s, 1H), 7.73 (d, *J*=7.7 Hz, 1H), 7.38 (d, *J*=8 Hz, 1H), 7.30 (t, *J*=7.5 Hz, 1H), 7.17 (t, *J*=6.4 Hz, 1H), 6.77 (s, 1H), 6.70 (d, *J*=7.4 Hz, 1H), 6.58 (d, *J*=7.8 Hz, 1H), 6.27 (s, 1H), 4.01-4.15 (m, 2H), 2.30 (s, 3H), 1.19 (t, *J*=7.1 Hz, 3H). IR (KBr): 3383, 2956, 2927, 1659, 1598, 1507, 1444, 1270, 1249, 1115, 1098, 752cm^{-1}. mp: 227-229 $^{\circ}$C.

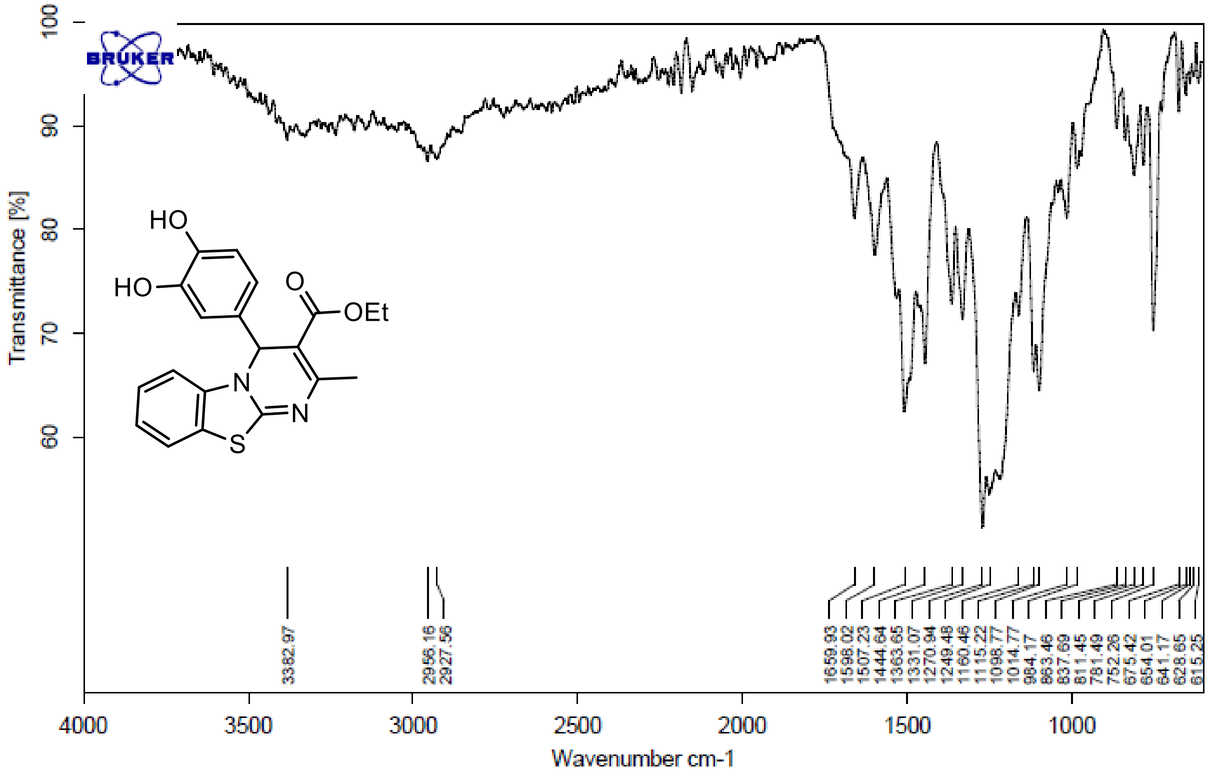

S37. The FT-IR spectrum of Ethyl-2-methyl-4-(3,4-dihydroxyphenyl)-4*H*-pyrimido[2,1-*b*][1,3]benzothiazole-3-carboxylate

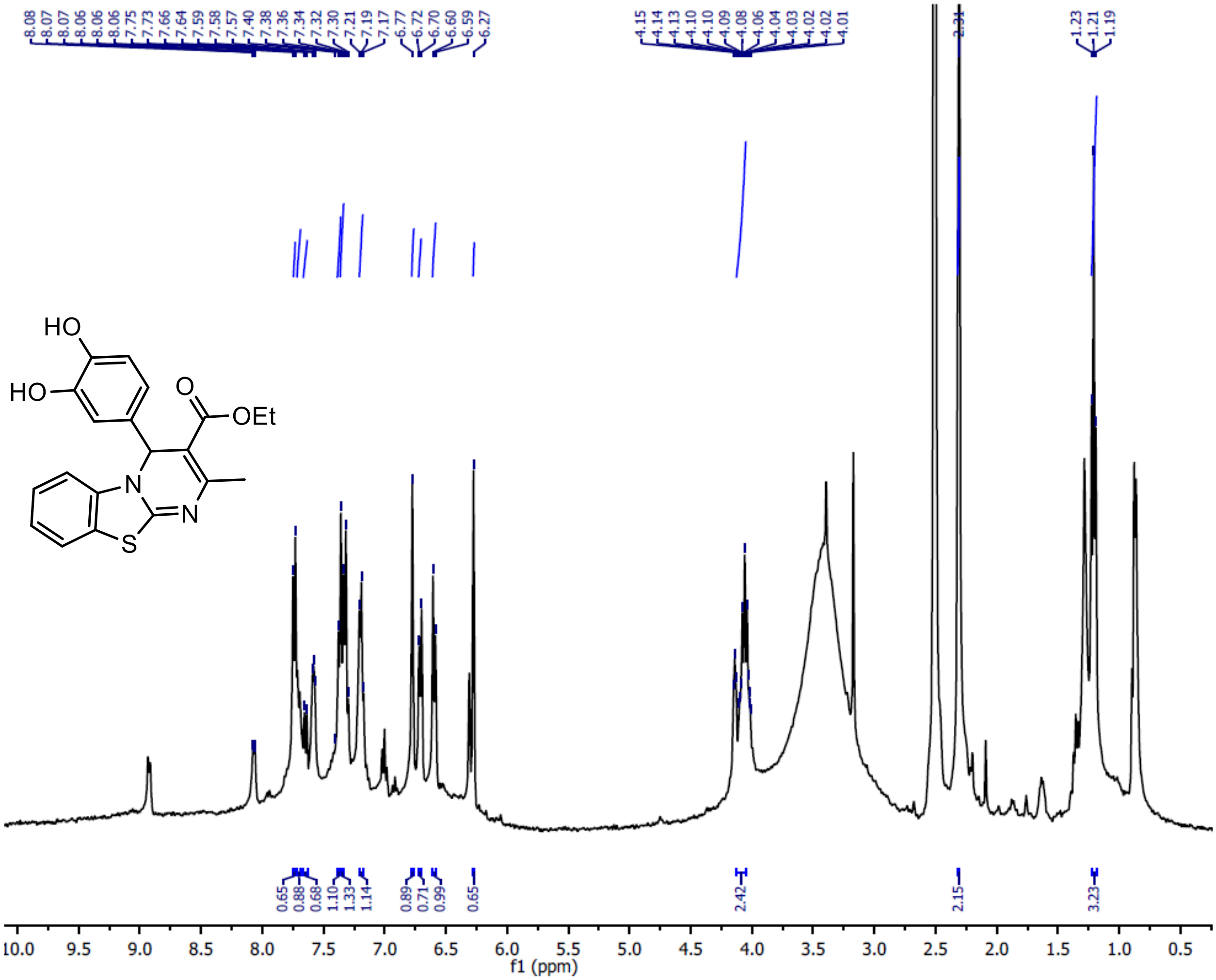

S38. The ^{1}H NMR (400MHz) spectrum of Ethyl-2-methyl-4-(3,4-dihydroxyphenyl)-4*H*-pyrimido[2,1-*b*][1,3]benzothiazole-3-carboxylate

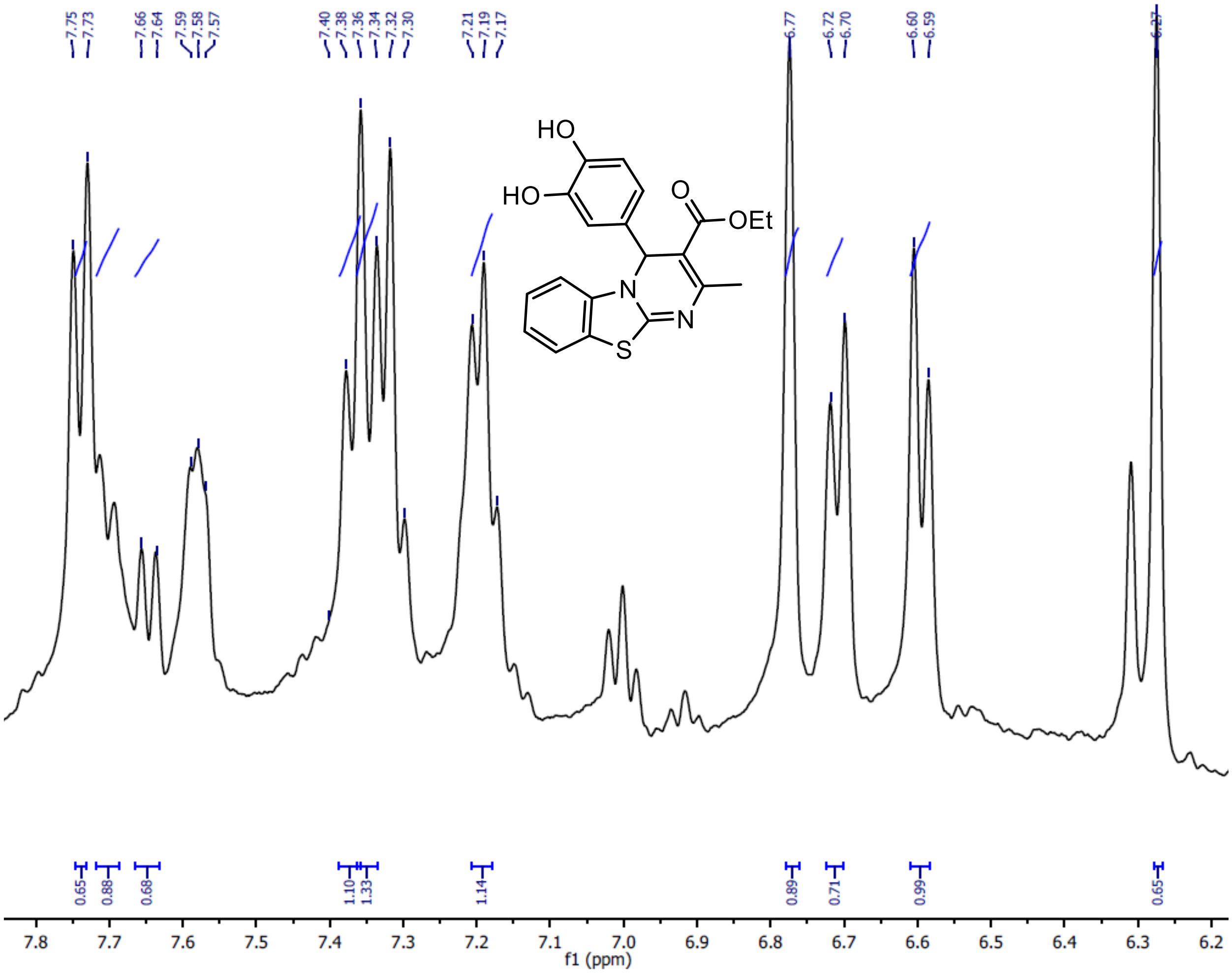

S39. The ^{1}H NMR (400MHz) spectrum of Ethyl-2-methyl-4-(3,4-dihydroxyphenyl)-4*H*-pyrimido[2,1-*b*][1,3]benzothiazole-3-carboxylate
